# RB1 controls differentiation through positive regulation of phosphoglycerate mutases

**DOI:** 10.1038/s41419-025-07850-3

**Published:** 2025-07-24

**Authors:** Susumu Kohno, Nobuyuki Okahashi, Yuansong Wan, Hai Yu, Yujiro Takegami, Paing Linn, Naoko Nagatani, Shunsuke Kitajima, Teruo Kawada, Fumio Matsuda, Hiroshi Shimizu, Chiaki Takahashi

**Affiliations:** 1 Cancer Research Institute, Kanazawa University, Kanazawa, Ishikawa, Japan; 2 Graduate School of Information Science and Technology, Osaka University, Suita, Osaka Japan; 3 Helen and Robert Appel Alzheimer’s Disease Institute, Feil Family Brain and Mind Research Institute, Weill Cornell Medicine, New York, NY USA; 4 DANAFORM, Tsurumi-ku, Yokohama, Kanagawa Japan; 5 Yangon General Hospital, Yangon, Lanmadaw Myanmar; 6 Japan Foundation for Cancer Research, Koto-ku, Tokyo Japan; 7 Laboratory of Molecular Function of Food, Graduate School of Agriculture, Kyoto University, Uji, Kyoto Japan

**Keywords:** Differentiation, Oncogenesis

## Abstract

Most glycolytic enzymes are transcriptionally controlled by hypoxia-inducible factor-1α (HIF-1α) and/or MYC, however, phosphoglycerate mutases (PGAMs) are exceptional. Retinoblastoma tumor suppressor 1 (RB1) loss converts poorly spherogenic *Trp53-*null leiomyosarcoma cells to highly spherogenic. We determined a gene expression signature of RB1 loss-of-function in this setting and identified PGAM2 as a positive transcriptional target of RB1. Later, we found that RB1 positively controls PGAM1 as well in different tissues. RB1 deficiency induced a metabolic shift in the glycolytic pathway in a manner compatible with PGAM downregulation. Many of the metabolic features induced by RB1 loss were antagonized by PGAM overexpression. Furthermore, differentiation deficiency following RB1 loss was rescued by PGAM overexpression or pyruvate supplementation to varied degrees. These findings suggest that the RB1-PGAM1/2 axis at least partially controls RB1-dependent differentiation.

## Introduction

The Cell cycle is tightly coupled to biomass synthesis. This predicts some of the cell cycle regulators might be concurrently involved in the metabolic control. RB1 tumor suppressor is primarily implicated in the control of G1 to S phase transition during the cell cycle [1]. However, an increasing amount of evidence indicates pivotal roles of RB1 in metabolic regulation [2–6]. RB1 controls mevalonate pathway through E2Fs and sterol regulatory element binding proteins (SREBPs) [7]. RB1 controls fatty acid elongation and desaturation through transcriptional regulation of elongation of long-chain fatty acids protein 6 (ELOVL6) and stearoyl-CoA desaturase 1 (SCD1) [8]. Additionally, RB1 has been linked to cytokine production by fatty acid oxidation and AMP-activated protein kinase (AMPK) in sarcoma and breast cancer [9, 10]. RB1 has been implicated in glutamine metabolism in drosophila and mammalian cells [2, 6]. In muscle, RB1 promotes mitochondrial function through inhibition of lysine demethylase 5 A (KDM5A) function [11]. In erythrocytes, RB1 has been linked to mitochondrial biogenesis [12]. In addition, in colon and lung cells, RB1 promotes mitochondrial function through mitochondrial translational proteins [13]. Inversely, RB1 suppresses mitochondrial function through E2Fs or peroxisome proliferator-activated receptorγ coactivator-1α (PGC-1α) in triple negative breast cancers and adipocytes [14, 15]. E2F1 mediates RB1 function to suppress oxidative phosphorylation (OXPHOS) in adipocytes [16, 17]. RB1 has been implicated in the control of glycolysis as well. E2Fs mediate RB1 function to enhance pyruvate dehydrogenase (PDH) function through transcriptional control of pyruvate dehydrogenase kinase 4 (PDK4) [18]. Lysine demethylase 4 A (KDM4A) cooperates with E2F1 to activate PDK1 and 3 [19]. In the context of cellular senescence, RB1 promotes the expression of a series of glycolytic genes [20]. These findings indicate that RB1 may impact on cellular state through its effect on cellular metabolism.

Because of its primary role in controlling the cell cycle, the mechanism of RB1 in differentiation control has been explained by the promotion of eternal exit from cell cycle. Additionally, RB1 cooperates with tissue-specific transcription factors such as MyoD, CCAAT/enhancer binding protein a (C/EBPα), nuclear factor for IL6 expression (NF-IL6), core binding factor 1 (CBFA1) or glucocorticoid receptor (GR), or antagonizes differentiation inhibitors such as inhibitor of DNA binding 2 (ID2), EP300 interacting inhibitor of differentiation 1 (EID1) or KDM5A to execute peripheral tissue development [4, 21]. RB1 inhibits the histone H3 at lysine 4 (H3K4) demethylase activity of KDM5A [22]. KDM5A may play a key role in coupling cellular metabolism to RB1-dependent differentiation control. Simultaneous inactivation of KDM5A rescued both oxygen consumption rate and differentiation defects in RB1-deficient myocytes [11]. In addition, shifts in mitochondrial biogenesis, autophagy or glycolysis may explain ineffective erythropoiesis or defective myogenic differentiation induced by RB1 loss [12, 23]. These findings indicate that the metabolic shift may account for differentiation defects associated with RB1-deficiency [3]. In this study, we focus on glycolytic enzyme PGAMs that appear to be positively regulated by RB1. Aerobic glycolysis supports developmental program of T cells [24]. Glycolysis governed by PGAM2 is essential for zebrafish muscle development [25]. Additionally, a mutation in the human PGAM2 gene causes myopathy with exercise intolerance [26]. These findings predicted that RB1 might control the differentiation program at least partially through transcriptional regulation of PGAMs.

In the current study, we initially sought to determine the RB1 loss signature in a *Trp53-*null leiomyosarcoma cell line. In this context, RB1 loss induces very high spherogenic activity with enhanced expression of a cancer stem-like cell and pluripotent stem cell markers, however, without much enhancing cell proliferation [9, 27]. In parallel, we determined metabolic features that appeared in the same RB1-inactivated cells using metabolome and extracellular flux analysis. These multi-omics studies identified PGAM2 as a molecule commonly depicted in both gene expression and metabolic signatures associated with RB1 loss. Later, we discovered that RB1 controls PGAM1 as well as PGAM2 in many cell lines. PGAMs constitute the lower part of the glycolytic pathway; they convert 3-phosphoglycerate (3-PG) to 2-phosphoglycerate (2-PG). Since this enzyme is not abundantly expressed [28], its downregulation can be rate-limiting in the glycolytic pathway. We thus expected that RB1 loss might affect glycolysis due to the downregulation of PGAMs. This study not only proves this idea but also highlights unexpected roles of the RB1-PGAMs axis in the behavior of stem-like cancer cells and in the RB1-dependent differentiation control of myocytes and adipocytes.

## Results

### Rb1 loss downregulates glycolytic activity and Pgam2 expression in mouse sarcoma

**Fig. 1:**
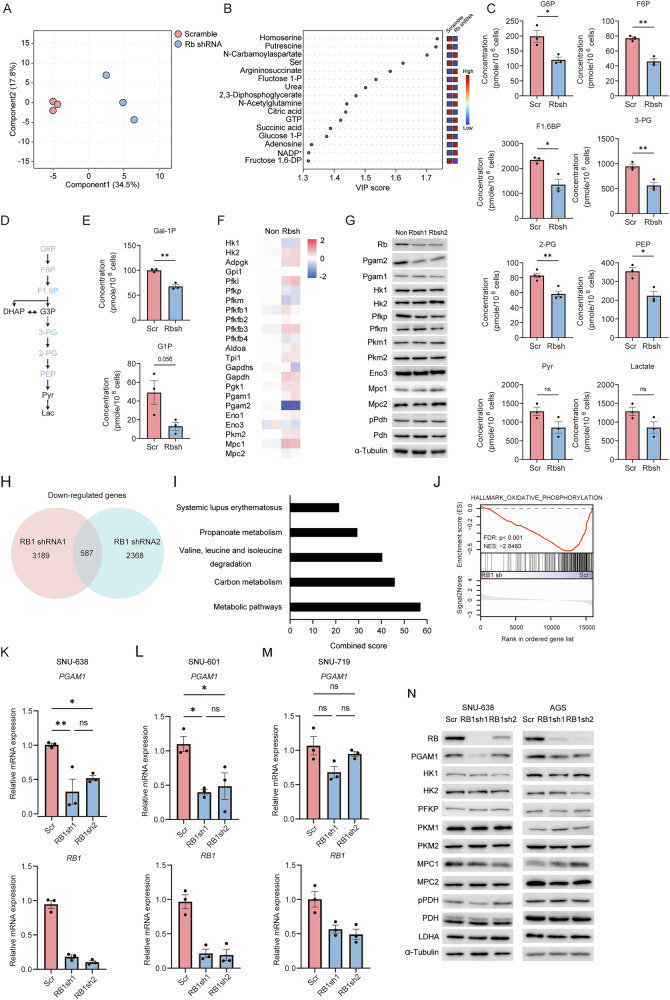
Impact of RB1 depletion on glycolysis in 53KOLS and gastric cancer cells. **A** The partial least-squares discriminant analysis (PLS-DA) of the metabolomic profile of Rbsh (knockdown; red dots) and control (blue dots) cells (*n* = 3). Detected metabolites in positive and negative ion modes of capillary electrophoresis-mass spectrometry (CE-MS) were quantified as standard curve. **B** The variable importance in projection (VIP) score of the top 15 metabolites that significantly distinguishes metabolic features of RB1-depleted cells from that of control cells. **C** Absolute concentration of metabolites in the glycolytic pathway. Data are shown as the mean ± standard deviation (SD). Two-tailed student’s t-test was performed. **P < 0.01, *P < 0.05 and n.s., not significant. **D** Illustration of the glycolytic pathway. Metabolites downregulated following Rb depletion are indicated in pale blue. **E** Absolute concentration of indicated metabolites. Data are shown as the mean ± standard deviation (SD). Two-tailed student’s t-test was performed. **P < 0.01 and n.s., not significant. **F** Heatmap of the expression of glycolytic genes before and after Rb depletion. **G** Immunoblotting (IB) of the indicated proteins in 53KOLS cells expressing scramble or Rb shRNA. **H** Venn diagram of down-regulated genes in RB1-depleted SNU-638 cells. Log Fc <-0.5, FDR < 0.12. **I** Combined score of pathway analysis conducted by Enrich R. **J** GSEA results for h.all.v7.0.symbols.gene.sets comparing SNU-638 cells expressing RB1 shRNA to those expressing scramble shRNA. **K**–**M** Relative mRNA expression levels of the indicated genes in RB1-depleted SNU-638 (**K**), SNU-601 (**L**), and SNU-719 (**M**) cells. Tukey’s honestly significant difference (HSD)-test was performed. **P < 0.01, *P < 0.05　and n.s., not significant. **N** IB of the indicated proteins in cells expressing scramble or RB1 shRNA. All data are shown as the mean ± SEM.

*TP53* mutation induces well-differentiated type sarcoma from human mesenchymal stem cells and additional mutation in *RB1* converts this to poorly differentiated pleomorphic type [29]. We recapitulated this story in a line of mice. We isolated cells from leiomyosarcoma developed in a *Trp53*^−/−^ C57BL/6 mouse [10, 27, 30], cultured them in dish for more than 10 passages, and named resultant cells 53KOLS. Additional deletion of Rb in this line did not induce accelerated cell proliferation but did induce the expression of a cancer stem cell marker aldehyde dehydrogenase 1A3 (ALDH1A3), and higher spherogenic activity [9]. As myofibroblasts exhibit increased glycolysis upon differentiation [31], we analyzed the central carbon metabolome in 53KOLS cells before and after Rb depletion. The partial least squares discriminant analysis (PLS-DA) using 101 metabolites revealed that several metabolites in 53KOLS cells were significantly accountable for the distinct metabolic states observed before and after Rb depletion (Fig. 1A). The variable importance projection (VIP) scoring indicated that fructose-1,6-diphosphate and 2,3-diphosphoglycerate are significantly responsible for the metabolic shift that appeared following Rb depletion (Fig. 1B). Further metabolome analysis detected a decrease in a series of glycolytic metabolites after Rb depletion (Fig. 1C, D) indicating that Rb status impacts the glycolysis in 53KOLS cells without giving a significant impact on their growth properties [9, 27]. Other hexose sugars such as galactose-1-phosphate (Gal1P) and glucose-1-phosphate (G1P) as well decreased in Rb-depleted 53KOLS cells (Fig. 1E). As there was no significant change in the expression level of smooth muscle cell-specific genes after Rb depletion (Supplementary Fig. S1A, B), dedifferentiation may not account for the downregulation of glycolysis.

We then focused on the expression of glycolytic genes in a transcriptome analysis. The expression level of PGAM2 was specifically downregulated in Rb-depleted cells (Fig. 1F). PGAM1 and 2 constitute the lower part of the glycolytic system by converting 3-PG to 2-PG. PGAM2 is the major isoform in muscle-related tissues, while PGAM1 is predominant in other tissue types. Consistent with the pathological diagnosis of leiomyosarcoma, 53KOLS cells expressed Pgam2 considerably higher than Pgam1. An immunoblotting (IB) analysis indicated that the expression of Pgam2 protein is specifically downregulated following Rb depletion (Fig. 1G). An RT-qPCR analysis demonstrated that Rb loss in 53KOLS cells significantly diminishes the transcription of Pgam2 (Supplementary Fig. S1C).

### RB1 loss decreases glycolysis and PGAM1 expression in gastric cancer

We next carried out gene set enrichment analysis (GSEA) to unearth any other types of common cancer that acquire lesser glycolytic phenotype due to downregulation of RB1. The enrichment plot of GSEA presented the down-regulated gene signature of both human gastric cancer and mouse gastric cancer models had significant correlation with that of Rb-depleted 53KOLS cells (Supplementary Fig. S2A, B). Gastric cancer has been classified into four subtypes including chromosomal instability (CIN), Epstein-Barr virus (EBV), genomically stable (GS), and microsatellite instability (MSI) [32]. The expression level of PGAM1 was significantly lower in CIN and GS than in other subtypes. This pattern was highly reminiscent of that of RB1 (Supplementary Fig. S2C, D), indicating that RB1 status might impact PGAM1 status or vice versa in gastric cancers.

We therefore analyzed gastric cancer cell lines by Cap-analysis of gene expression (CAGE) [33]. We focused on 587 genes downregulated commonly by RB1 shRNA1 and 2 in the SNU-638 RB1-positive gastric cancer cell line (Fig. 1H). Pathway analysis by enrichR identified glycolytic metabolism and central carbon metabolism as a positive transcriptional target of RB1 in SNU-638 cells (Fig. 1I). Most importantly, the glycolytic gene set in carbon metabolism responsible for a higher score in enrichR included PGAM1 (Fig. 1I). Also, genes related to OXPHOS were enriched in control cells when compared to RB1-depleted (Fig. 1J). Indeed, the expression of PGAM1 was downregulated following RB1 depletion in SNU-638 and SNU-601 RB1-positive gastric cancer cell lines however, not in SNU-719 in which RB1 protein has been already inactivated by the product of EBV (Fig. 1K–M). An IB analysis demonstrated that PGAM1 downregulation following RB1 depletion in SNU-638 and AGS cells is quite specific in glycolytic enzymes (Fig. 1N).

### RB1 loss decreases glycolytic flux

**Fig. 2:**
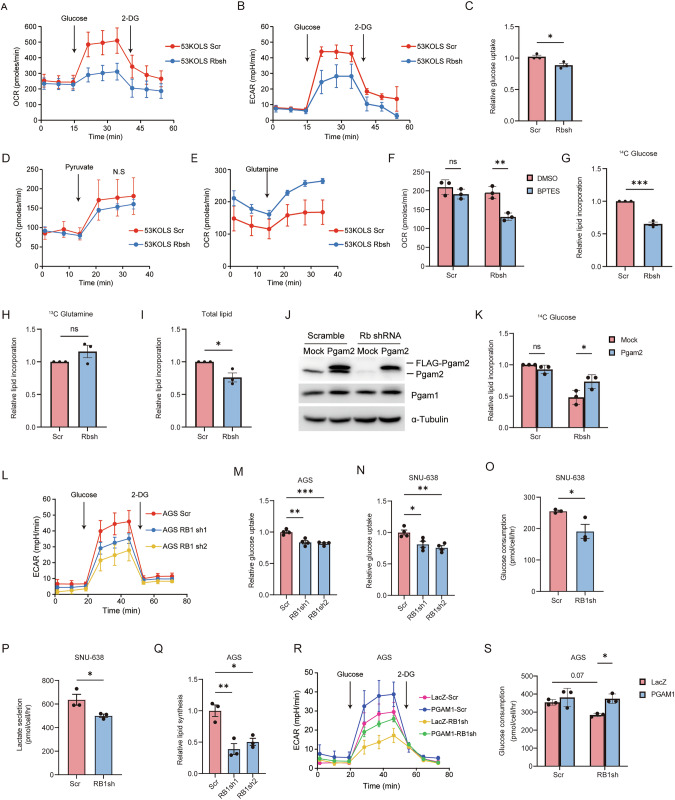
RB1 depletion reduces glucose-derived carbon entry into TCA cycle in 53KOLS and gastric cancer cells. **A** OCR, an index of oxygen consumption in mitochondria indicating oxidative phosphorylation coupled with glycolytic activity, was measured in 53KOLS cells expressing scramble or Rb shRNA. Cells were sequentially treated with 10.5 mM glucose and 100 mM 2-deoxy glucose. **B** ECAR, an index of the rate of lactate production indicating glycolytic activity, was measured in 53KOLS cells expressing scramble or Rb shRNA. Cells were sequentially treated with 10.5 mM glucose and 100 mM 2-deoxy glucose (2-DG). **C** Relative 2-(N-(7-Nitrobenz-2-oxa-1,3-diazol-4-yl)Amino)-2-Deoxyglucose (2-NBDG) fluorescence intensity showing glucose uptake in 53KOLS cells expressing scramble or Rb shRNA. Paired student’s t-test was performed. *P < 0.05　and n.s., not significant. **D** ECAR was measured in 53KOLS cells expressing scramble or Rb shRNA following the treatment with 1 mM pyruvate. **E** ECAR was measured in 53KOLS. cells expressing scramble or Rb shRNA following the treatment with 4 mM glutamine. **F** OCR in 53KOLS cells expressing scramble or Rb shRNA was measured in the presence or absence of a glutaminase inhibitor, BPTES (5 μM). Tukey HSD test was performed. **P < 0.01 and n.s., not significant. **G** Relative incorporation of [U-^14^C]-glucose into lipid. 53KOLS cells were cultured in the presence of [U-^14^C]-glucose (6.25 μCi/ml) for 24 h. ^14^C activity was measured using liquid scintillation counter. Paired student’s t-test was performed. ***P < 0.001. **H** Relative incorporation of [U-^14^C]-glutamine into lipid. 53KOLS cells were cultured in the presence of [U-^14^C]-glutamine (62.5 μCi/ml) for 24 h. Paired student’s t-test was performed. n.s., not significant. **I** Relative lipid incorporation of ^3^H_2_O. 53KOLS cells were cultured in the presence of ^3^H_2_O (20 v/v%) for 24 h. ^3^H activity was measured using liquid scintillation counter. Paired student’s t-test was performed. *P < 0.05. **J** IB of the indicated proteins in 53KOLS cells overexpressing Pgam2. **K** Relative lipid incorporation of [U-^14^C]-glucose in 53KOLS cells overexpressing Pgam2. Tukey’s HSD test was performed. *P < 0.05 and n.s., not significant. **L** ECAR was measured in AGS cells expressing scramble or RB1 shRNA. Cells were sequentially treated with 10.5 mM glucose and 100 mM 2-DG. **M** Relative 2-NBDG fluorescence intensity in AGS cells expressing scramble or RB1 shRNA. Tukey HSD test was performed. ***P < 0.001 and **P < 0.01. **N** Relative 2-NBDG fluorescence intensity in SNU-638 cells expressing scramble or RB1 shRNA. Tukey HSD test was performed. **P < 0.01 and **P < 0.05. **O** Absolute glucose consumption rate within 24 h in SNU-638 cells expressing scramble or RB1 shRNA. Paired student’s t-test was performed. *P < 0.05. **P** Absolute lactate secretion rate within 24 h in SNU-638 cells expressing scramble or RB1 shRNA. Paired student’s t-test was performed. *P < 0.05. **Q** Relative incorporation of [U-^14^C]-glucose into lipid in AGS cells expressing scramble or RB1 shRNA those were cultured in the presence of [U-^14^C]-glucose (6.25 μCi/ml) for 24 h. ^14^C activity was measured using liquid scintillation counter. Tukey HSD test was performed. **P < 0.01 and *P < 0.05. **R** Effect of PGAM1 overexpression on glycolytic flow in RB1-depleted AGS cells. **S** Effect of PGAM1 overexpression on glucose consumption for 24 h in RB1-depleted AGS cells. Glucose concentration in medium was determined using colorimetric assay. Tukey HSD test was performed. *P < 0.05. All data are shown as the mean ± SEM.

Next, we addressed whether RB1 deficiency causes any change in the use of carbon sources. We assessed oxygen consumption rate (OCR) and extracellular acidification rate (ECAR) after glucose starvation and restimulation. Rb depletion in 53KOLS cells decreased both OCR and ECAR upon glucose stimulation (Fig. 2A, B). Glucose uptake was significantly suppressed by Rb depletion in 53KOLS (Fig. 2C). When pyruvate was supplied at 2 mM, Rb depletion induced no significant changes in OCR in 53KOLS cells (Fig. 2D), indicating that the effect of reduced glycolysis on mitochondrial activity is counterbalanced by another metabolic flux from pyruvate. As literary expected from the role of RB1 in glutamine metabolism [2, 6], we detected increased OCR in response to glutamine stimulation following Rb depletion (Fig. 2E). These findings indicate that downregulated glycolysis and upregulated glutamine metabolism are characteristic of the metabolic shift induced by RB1 loss. Consistent with this view, the basal OCR did not differ between control and Rb-depleted 53KOLS cells; however, Rb-depleted cells appeared to be more sensitive to bis-2-(5-phenylacetamido-1,2,4-thiadiazol-2-yl)ethyl sulfide (BPTES) which suppresses glutamine metabolism by inhibiting glutaminase (GLS) activity (Fig. 2F). To further confirm decreased glycolysis following Rb loss, we measured ^14^C incorporation into de novo synthesized fatty acid. As expected, glucose-derived ^14^C was less incorporated into fatty acid following Rb loss (Fig. 2G). However, the uptake of glutamine-derived ^14^C did not significantly change following Rb loss (Fig. 2H). The degree of decrease in glucose that engages into fatty acid flow was compatible with that in de novo fatty acid synthesis estimated by ^3^H_2_O incorporation (Fig. 2I). These findings suggest that RB1 loss directly affects glucose metabolism rather than glutamine metabolism.

As aforementioned, glycolytic genes PGAMs are specifically downregulated following RB1 loss; this may account for the downregulated glycolysis induced by RB1 loss. We introduced FLAG-tagged Pgam2 into Rb1-depleted 53KOLS cells (Fig. 2J); this significantly antagonized the effect of Rb depletion on ^14^C incorporation into de novo synthesized fatty acid (Fig. 2K). All these findings suggested that Rb loss decreases glycolytic flux in 53KOLS cells.

We subsequently analyzed gastric cancer cell lines using the XF24 analyzer. Glycolytic activity was assessed using the same method as 53KOLS. AGS and SNU-638 cells exhibited reduced ECAR following RB1 depletion (Fig. 2L, Supplementary Fig. S3A). Consistent with these findings, glucose uptake was diminished in RB1-depleted gastric cancer cells (Fig. 2M, N). Additionally, glucose consumption in the culture medium was reduced in RB1-depleted SNU-638 cells (Fig. 2O). This aligns with the observation of decreased lactate secretion in SNU-638 cells (Fig. 2P). To further confirm the reduction in glycolysis following RB1 loss, we measured ^14^C-glucose incorporation into de novo synthesized fatty acids. Indeed, RB1 depletion led to a decrease in glucose-derived carbon in de novo synthesized fatty acids (Fig. 2Q, Supplementary Fig. S3B). The pentose phosphate pathway, which branches from the upper part of glycolysis, supports de novo DNA synthesis. We found that ^14^C-glucose incorporation into nuclear DNA was not affected by RB1 depletion, which suggests that carbon flow through the upper part of glycolysis is not affected by RB1 status (Supplementary Fig. S3C). RB1 loss downregulates PGAM1 expression in gastric cancer cells as shown in Fig. 1. Introduction of tagged-PGAM1 into RB1-depleted AGS cells significantly antagonized the effects of RB1 depletion on ECAR and de novo synthesized fatty acids (Fig. 2R, S). All these findings suggest that RB1 loss decreases glycolytic flux and that RB1 controls glycolysis at least partially through transcriptional regulation of PGAMs.

### RB1 loss does not affect mitochondrial function

We then focused on an RB1-positive gastric cancer AGS cells because this line is adhesive to the dish and much easier to observe cell morphology and organelles upon imaging compared to SNU-638 or SNU-601. We next assessed whether the mitochondrial status is affected by RB1 loss in cell lines we employed. The ratio of mitochondrial gene versus nuclear gene (mitochondrial copy number) changed neither in 53KOSL nor AGS gastric cancer cells following Rb/RB1 depletion (Supplementary Fig. S4A, B). The fluorescence intensity of MitoTracker, which is a mitochondrial surface anchor dye, was unaffected in Rb/RB1-depleted 53KOLS nor in AGS cells (Supplementary Fig. S4C, D). Fluorescence intensity of tetramethylrhodamine methyl ester (TMRM), which is an indicator of mitochondrial membrane potential, was affected by Rb/RB1 loss neither in 53KOLS nor AGS cells (Supplementary Fig. S4E, F). In addition, the mitostress test exhibited that Rb/RB1 loss affected coupled respiration and maximum respiration capacity neither in 53KOLS nor AGS cells (Supplementary Fig. S4G, H). These data suggested that Rb/RB1 status does not affect mitochondrial status at least in the cells we tested in this study.

### RB1 controls sphere formation through PGAM regulation

**Fig. 3:**
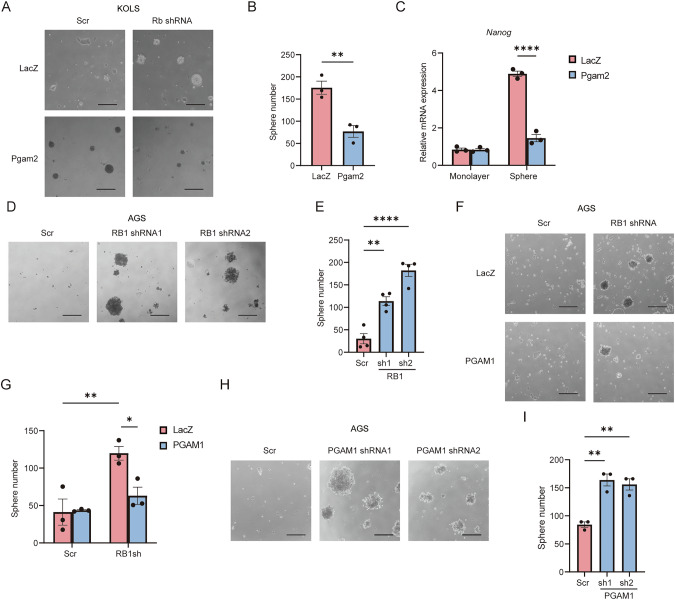
Loss of PGAM induces sphere formation. **A** Phase-contrast image of spheres developed from Rb-depleted 53KOLS cells expressing LacZ or PGAM2. **B** Number of spheres developed from Rb-depleted 53KOLS cells expressing LacZ or PGAM2. **C** mRNA expression of the indicated gene in monolayer or spheroid. Tukey’s HSD test was performed. ****P < 0.0001. **D** Phase-contrast image of spheres developed from AGS cells expressing scramble or RB1 shRNA. Scale bar; 200 μm. **E** Number of spheres developed from RB1-depleted AGS cells under 3D culture conditions. Tukey’s HSD test was performed. **P < 0.01 and *P < 0.05. **F** Phase-contrast image of spheres developed from AGS cells expressing LacZ or PGAM1, those with or without RB1-depletion. **G** Number of spheres developed from the indicated cells cultured under the indicated 3D conditions. **H** Phase-contrast image of spheres developed from AGS cells expressing scramble or PGAM1 shRNA. **I** Number of spheres developed from PGAM1-depleted AGS cells under 3D culture conditions. Tukey’s HSD test was performed. **P < 0.01. All data are shown as the mean ± SEM.

Rb loss induces the formation of undifferentiated and spherogenic cells in an interleukin-6 (IL6)-dependent manner in 53KOLS cells [9, 27]. Homozygous PGAM1 loss is embryonic lethal at embryonic day 13.5, suggesting that PGAM1 is essential for the development of embryos and maintenance of an undifferentiated state [34]. We therefore addressed whether the RB1-PGAM axis controls spherogenesis in 53KOLS cells. As we reported previously, Rb depletion enhanced spherogenesis in 53KOLS cells, which was significantly antagonized by the introduction of Pgam2 (Fig. 3A, B). Rb-depleted spheroid cells expressed significantly higher levels of Nanog compared to monolayer cultures in 53KOLS. Notably, Pgam2 overexpression antagonized this effect of Rb depletion (Fig. 3C). These findings indicate that Rb loss induces an undifferentiated state through the downregulation of Pgam2 expression. RB1 depletion in AGS gastric cancer cells as well enhanced spherogenesis, exactly as observed in 53KOLS cells (Fig. 3D, E). The introduction of PGAM1 antagonized this effect of RB1 depletion (Fig. 3F, G). Surprisingly, PGAM1 depletion increased sphere formation in gastric cancer cells even without RB1 depletion (Fig. 3H, I). These data suggest that the reduction of PGAM expression is crucial for sphere formation and induction of an undifferentiated state.

### RB1 controls myogenic differentiation through PGAM2 regulation

**Fig. 4:**
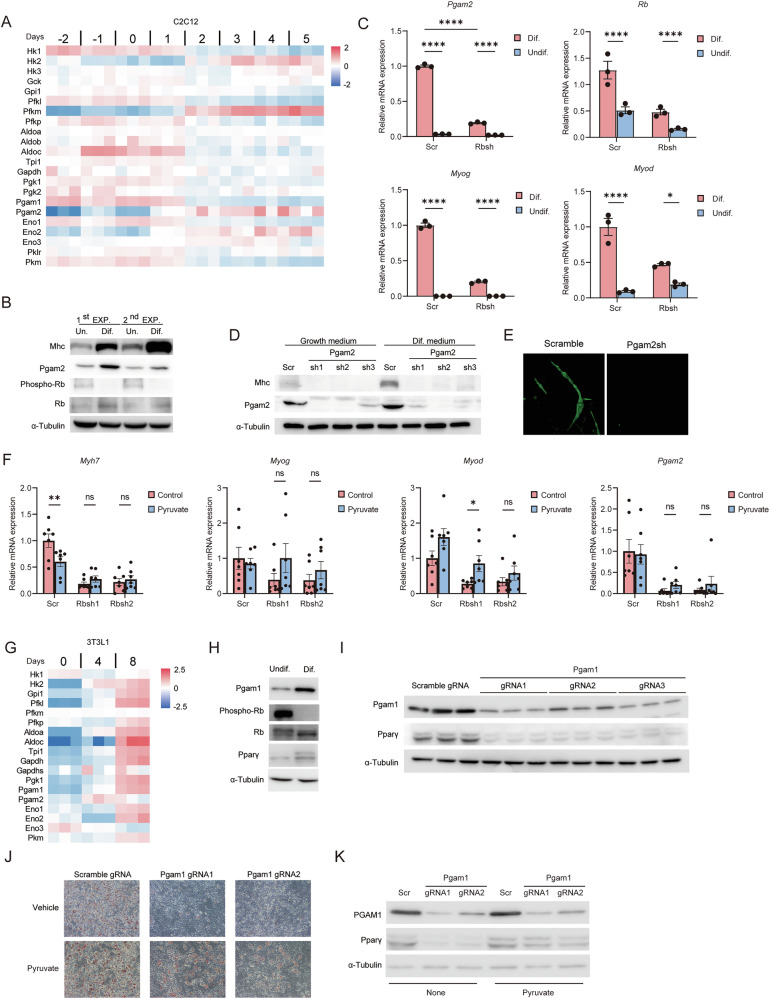
Induction of PGAM is essential for myogenic and adipogenic differentiation. **A** Heatmap of genes related to glycolysis in a time course microarray sampled during differentiation in C2C12 cells. **B** IB of the indicated proteins in differentiated and undifferentiated C2C12 cells. **C** mRNA expression levels of the indicated genes in C2C12 cells expressing scramble or Rb shRNA. C2C12 cells were differentiated under low serum conditions for 7 days. Tukey’s HSD test was performed. ****P < 0.0001 and *P < 0.05. **D** IB of the indicated proteins in C2C12 cells expressing scramble or Pgam2 shRNA those with or without exposure to stimuli to induce myogenic differentiation. **E** Immunocytochemistry of myosin heavy chain (MHC) in C2C12 cells expressing scramble or Pgam2 shRNA and exposed to stimuli to induce myogenic differentiation for 7 days. **F** mRNA expression levels of the indicated genes in C2C12 cells expressing scramble or Rb shRNA. Cells were differentiated in the presence or absence of pyruvate for 7 days. Tukey’s HSD test was performed. **P < 0.01, *P < 0.05 and n.s.; not significant. **G** RNA-seq heatmap of genes related to glycolysis during adipogenic differentiation in 3T3L1 cells. **H** IB of the indicated proteins in differentiated and undifferentiated 3T3L1 cells. **I** IB of the indicated proteins in PGAM1-depleted 3T3L1 after exposure to stimuli to induce adipogenic differentiation. **J** Oil red staining of 3T3L1 cells transduced with the indicated gRNA and treated with vehicle or 4 mM pyruvate under the culture conditions to induce adipogenic differentiation. **K** IB of the indicated proteins in PGAM1-depleted 3T3L1 cells after exposure to stimuli to induce adipogenic differentiation in the presence or absence of 4 mM pyruvate. All data are shown as the mean ± SEM.

RB1 has been thought to control a series of differentiation programs primarily through its influence on cell cycle and tissue-specific transcription factors. Adjustment of energy production may constitute another mechanism of RB1 in controlling differentiation programs. We addressed whether glycolytic genes are induced to meet energy demands during myogenic differentiation by analyzing time-course microarray data of C2C12 myoblast cells cultured under differentiation-inducing conditions. Hexokinase 2 (Hk2), 6-phosphofructokinase (Pfkm) and Pgam2 were clearly induced during myogenic differentiation (Fig. 4A). Importantly, we observed that Pgam2 induction coincided with myosin heavy chain (MHC) induction and RB1 hypophosphorylation (Fig. 4B). RB1 inactivation is known to abort myogenic differentiation program in C2C12 cells [35]. Notably, Rb loss strongly antagonized Pgam2 induction in C2C12 cells that were forced to differentiate (Fig. 4C). Moreover, Pgam2 depletion almost completely abrogated MHC induction and myotube formation upon stimulation to trigger myogenic differentiation (Fig. 4D, E). These findings indicate that Pgam2 is required for Rb-dependent myogenic differentiation.

As well known, Rb loss in C2C12 cells abrogates the induction of both early (MyoD and myogenin) and late (Myh7) markers of myogenic development (Fig. 4F). Pyruvate is the end-product of glycolysis. We demonstrated that supplementation of excessive pyruvate rescued MyoD and myogenin but not Myh7 even when Rb was depleted (Fig. 4F, Supplementary Fig. S5A). These findings indicate that Rb controls early phase of myogenic differentiation at least partially through Pgam2 regulation.

### RB1 controls adipogenic differentiation through PGAM1 regulation

RB1 promotes adipocyte differentiation by inducing cell cycle exit and enhancing the transactivation of adipogenic CCAAT/enhancer binding proteins (C/EBP) [36]. We inferred that glucose-derived carbon supply to the citric acid (TCA) cycle via PGAM is important even in adipogenic differentiation. 3T3L1 cells turned oil red positive and expressed PPARγ after MDI (3-isobutyl-1-methylxanthine: IBMX, dexamethasone: DEX, and insulin) treatment. Very importantly, this adipogenic conversion was significantly associated with upregulated expression of Pgam1 (Fig. 4G, H). Additionally, the depletion of Pgam1 significantly decreased the expression of PPARγ and the number of oil-red positive cells (Fig. 4I, J). Like in the case of myogenesis, the addition of pyruvate partially but significantly rescued oil red positive population and PPARγ expression in Pgam1tdepleted 3T3L1 cells (Fig. 4J, K). These findings indicate that RB1 controls adipogenic differentiation at least partially through PGAM1 regulation.

### MEF2s are involved in the transcriptional regulation of PGAM2

**Fig. 5:**
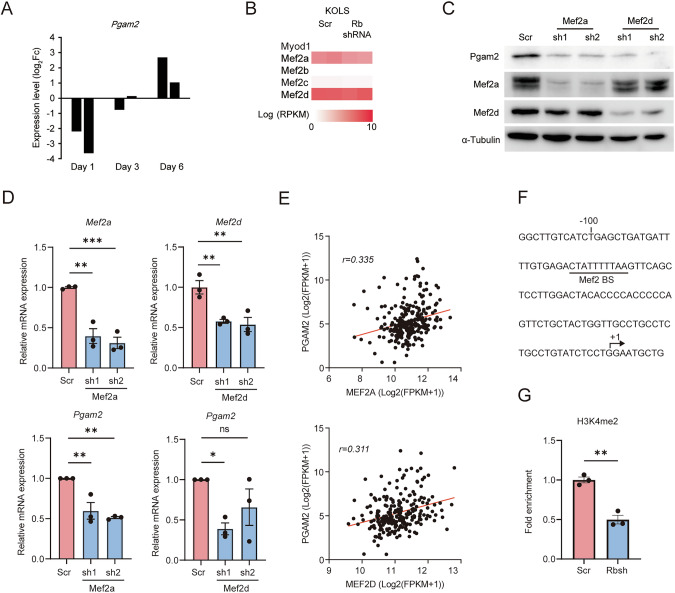
Mef2s regulate Pgam2 expression in 53KOLS cells. **A** Expression levels of Pgam2 in Myod-depleted C2C12 cells exposed to stimuli to induce myogenic differentiation. Expression levels of Pgam2 from microarray were shown in log_2_Fc. **B** Expression levels of myogenic transcription factors in 53KOLS cells. Data from RNA-seq was shown in Log_2_RPKM. **C** IB of the indicated protein in Mef2a or Mef2d-depleted 53KOLS cells. **D** Relative mRNA levels of the indicated genes in 53KOLS cells expressing scramble, Mef2a, or Mef2d shRNA. Data are shown as the mean ± standard error (SEM). Tukey HSD test was performed. ***P < 0.001, **P < 0.01, *P < 0.05 and n.s., not significant. **E** Correlation of mRNA expression level between MEF2A/D and PGAM1 genes in TCGA dataset. Spearman’s correlation test was performed. **F** Sequence of the promoter region of mouse Pgam2 gene. **G** ChIP-qPCR assessment of H3K4me2 at the Mef2 binding site in the Pgam2 promoter of 53KOLS cells, those with or without Rb depletion. Paired student’s t-test was performed. **P < 0.01. All data are shown as the mean ± SEM.

We sought the mechanism whereby PGAM2 is transcriptionally upregulated by RB1. MyoD has been implicated in RB1-dependent myogenic differentiation control [37] however, previous literature indicated that the E box motif (possible MyoD binding site) found in the Pgam2 promoter region was dispensable for transcriptional control [38]. Indeed, MyoD-depleted C2C12 cells were able to induce Pgam2 expression when exposed to stimuli to induce myogenic differentiation (Fig. 5A), and 53KOLS cells do not express MyoD (Fig. 5B). Furthermore, TP53 has been implicated in the transcriptional control of PGAM2 [39]. However, 53KOLS cells lack Trp53. These findings indicated that neither MyoD nor p53 participates in the mechanism whereby RB1 controls PGAM2 expression.

We then found Nakatsuji et al. indicated that MEF2 binding sites are critical for PGAM2 promoter activity [38]. Indeed, depletion of Mef2a or Mef2d in 53KOLS cells significantly suppressed the expression of Pgam2 (Fig. 5C, D). TCGA dataset in soft tissue sarcoma showed that PGAM2 expression positively correlates with MEF2A and MEF2D expression (Fig. 5E). We finally found that depletion of Rb reduces H3K4 dimethylation of the Mef2 binding site in the Pgam2 promoter (Fig. 5F, G). These findings indicate that RB1 promotes MEF2A/D recruitment to PGAM2 promoter in myocytes and adipocytes, thus enhances glycolytic activity required for myogenic and adipogenic differentiation.

### RB1-KDM5A axis controls PGAM1 transcription

**Fig. 6:**
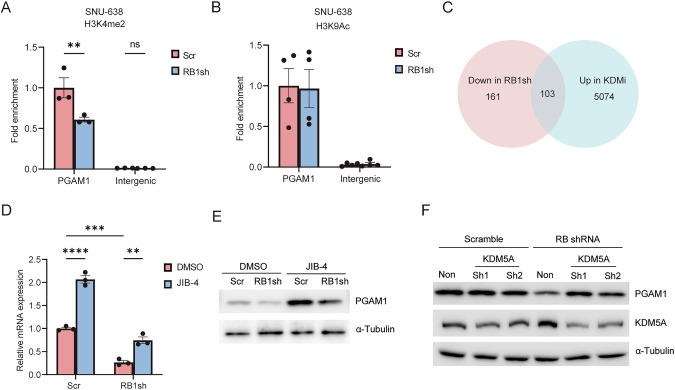
KDM5A regulates PGAM1 expression in gastric cancer cells. **A** H3K4me2 ChIP-qPCR of PGAM1 and intergenic regions in SNU-638 cells those transduced with scramble or RB1 shRNA. Tukey’s HSD test was performed. **P < 0.01, and n.s., not significant. **B** H3K9Ac ChIP-qPCR of PGAM1 and intergenic regions in SNU-638 cells those transduced with scramble or RB1 shRNA. Tukey’s HSD test was performed. n.s., not significant. **C** Venn diagram of number of genes that were down-regulated in RB1-depleted and upregulated in RB1-depleted SNU-638 cells upon treatment with a KDM inhibitor. **D** Relative mRNA levels of PGAM1 in RB1-depleted SNU-638 cells upon treatment with 1 mM JIB-4 or DMSO. Tukey’s HSD test was performed. ****P < 0.0001, ***P < 0.001 and **P < 0.01. **E** IB of the indicated proteins in SNU-638 cells expressing scramble or RB1 shRNA and treated with 1 mM JIB-4 or DMSO. **F** IB of the indicated proteins in RB1-depleted cells expressing scramble or KDM5A shRNA. All data are shown as the mean ± SEM.

RB1 interacts with various epigenetic modulators such as histone acetyltransferase or histone demethylase. KDM5A is one of the proteins that physically interact with RB1. Loss of RB1 expression liberates and thereby activates KDM5A, leading to a reduction in H3K4 methylation levels. Indeed, RB1 depletion reduced H3K4me2 levels in the promoter of PGAM1 in SNU-638 cells (Fig. 6A). In contrast, RB1 depletion did not affect histone acetylation in the promoter of PGAM1 of the same gastric cancer cells (Fig. 6B). We then investigated whether RB1 controls PGAM1 expression through KDM5A in gastric cancer cells. To elucidate the transcriptional regulation of RB1 via KDM5A, we conducted native elongating transcript cap analysis of gene expression (NET-CAGE) to analyze KDM5A-regulated genes in RB1-depleted gastric cancer cells. NET-CAGE is a technology that maps the positions of transcription start sites on the genome at single-nucleotide resolution using the CAGE method after enriching nascent RNA strands in the nucleus [40]. Thus, NET-CAGE enables real-time gene expression analysis based on the transcriptional activity of RNA Pol II products during synthesis. The result showed that 103 genes, including PGAM1, were specifically regulated by the RB1-KDM5A axis in gastric cancer cells (Fig. 6C). Importantly, the reduced expression of PGAM1 in RB1-depleted cells was restored by a KDM5A inhibitor or shRNA targeting KDM5A (Fig. 6D–F). KDM5A removes histone methylation by dissociating from RB1. These results indicate that the RB1-KDM5A axis controls PGAM1 expression in gastric cancer cells.

## Discussion

Metabolic reprogramming is frequently observed during the differentiation of various types of tissues and immune cells, such as muscle, adipocytes, or T cells [41–45]. For instance, muscle stem cells undergo a dramatic metabolic switch to oxidative phosphorylation during differentiation, associated with a massive increase in mitochondrial activity [46]. The demand for energy sources that increases in differentiated cells is typically met by remodeling in central carbon metabolism. Indeed, high glucose uptake is required during the first three days of differentiation to ensure proper maturation of adipocytes [47]. Additionally, glucose restriction inhibits myoblast differentiation by activating the AMPK-sirtuin 1 (SIRT1) pathway [48]. In this study, we found that RB1-dependent induction of PGAM redirects carbon flow during myogenic and adipogenic differentiation. Increased glucose flux upregulates nicotinamide metabolism to repress the nicotinamide adenine dinucleotide (NAD^+^)-dependent enzyme SIRT1, which promotes the commitment of adipocyte and muscle differentiation [47–49]. Inversely, PGAM depletion has been shown to prevent differentiation by increasing the NAD^+^/ NADH ratio [50]. Rapidly proliferating cells exhibit a metabolic phenotype known as aerobic glycolysis, which is defined by an increased rate of lactate production even in the presence of a sufficient level of oxygen. During glycolysis, glyceraldehyde-3-phosphate dehydrogenase (GAPDH) produces NADH from NAD^+^, while lactate dehydrogenase (LDH) or malate dehydrogenase 1 (MDH1) consumes NADH, consequently balancing the NAD^+^/NADH ratio in proliferating cells [51, 52]. Stimulation to induce cellular differentiation provokes an increase in OXPHOS activity. This reduces lactate production and LDH activity, resulting in an elevation of the NADH/NAD^+^ ratio [48, 53, 54]. Furthermore, acetylation on PGAM enhances its activity, whereas SIRT1-mediated deacetylation reduces it [55]. SIRT1 thus antagonizes PGAMs functions to promote differentiation. These findings suggest that RB1 loss depletes PGAM expression, potentially leading to alterations in nicotinamide metabolism and consequently interfering with differentiation.

Two isoforms PGAM1 and PGAM2 are differentially expressed in tissues. PGAM1 is dominantly expressed in the brain, kidney, and liver, while PGAM2 is expressed mainly in the heart, muscle, and testis [56, 57]. PGAM2 is specifically induced during myogenesis and spermatogenesis [58, 59]. A mutation in PGMA2 impedes myogenic differentiation by preventing the sumoylation on it and therefore reducing its activity [60]. A deficiency in PGAM2 in muscles leads to a metabolic myopathy known as glycogen storage disease X [26]. Interestingly, in muscle of patients with PGAM2 deficiency, PGAM1 is dominantly expressed instead of PGAM2 [26]. This indicates that specific expression of PGAM2 is crucial for maintaining proper muscle function which cannot be alternated by PGAM1. On the other hand, homozygous knockout of Pgam1 in mice results in embryonic lethality, highlighting its essential role in development [34]. PGAM1 deficiency shifts cell fate from white to brown or beige adipocytes, indicating its role in adipocyte differentiation [61]. Similarly, conditional knockout of Pgam1 in mouse T cells reduces cytokine responses, which affects differentiation [50]. From another perspective, RB1 cooperates with tissue-specific transcription factors to drive terminal differentiation in muscle and adipocytes [36, 62–65]. Retinoblastoma family proteins function as molecular switches that determine the differentiation of white and brown adipocytes [15, 66]. The fate decision governed by PGAMs in muscle and adipose tissue has been known to be achieved within the RB1-dependent context. Our findings strongly suggest that RB1 governs PGAMs expression during differentiation. Beyond its roles in regulating cell cycle, transcription factors, or chromatin modifiers, RB1 primarily modulates metabolism and thereby plays a critical role in supporting tissue-specific cell differentiation systems.

Rapidly proliferating cells need to acquire substantial amounts of building blocks such as nucleotides, proteins, or lipids. For instance, DNA synthesis necessitates nucleotide synthesis during the S phase. The components of nucleotides include sugar, glutamine, aspartate, glycine, or N-formyl-tetrahydrofolate (THF). To meet demand, the supply of these components increases during the S phase depending on the activation of GLS, transketolase-like-1 (TKTL1), glucose-6-phosphate dehydrogenase (G6PDH), or aldolase (ALDO) [67–71]. In contrast, during the G1-S transition, glycolytic activity decreases due to the downregulation of HK2 and 6-phosphofructo-2-kinase/fructose-2,6-biphosphatase 3 enzyme (PFKFB3) expression [71–74]. HK2 is a transcriptional target of cyclin D1, suggesting that the downregulation of glycolysis consists one of the mechanisms that ensure G1-S check point [75]. Throughout the entire G1-G2 phase, an increase in cell size dilutes RB1 concentration, which at the end of G2 phase triggers cell division [76]. This most probably implies that the cell cycle is tightly coupled with metabolism. However, cyclin-dependent kinase 4 and 6 (CDK4/6) inhibition did not increase PGAM expression (data not shown), nor is PGAM targeted by E3 ligases such as Skp1-Cullin 1-F-box (SCF) and anaphase-promoting complex (APC) those typically employed to achieve cell cycle-dependent build and scrap. Most of the glycolytic genes, except for Pgam2 and Pfkm, are downregulated during myogenic differentiation (Fig. 4A). These data suggest that the mode of regulation of differentiation via the RB1-PGAM axis is distinct from that of cell cycle regulation via the RB1-E2F axis.

The oncogenic transcription factors MYC and HIF-1α critically regulate cellular metabolism in cancer by driving metabolic reprogramming that supports tumor growth and survival [77, 78]. MYC, as a key driver of proliferation, upregulates pathways such as glutaminolysis, lipid biosynthesis, or glycolysis to meet the heightened demand for macromolecular precursors necessary for rapid cell division [77]. Under hypoxic conditions, where oxygen availability is limited, cells switch from oxidative phosphorylation to glycolysis for adenosine triphosphate (ATP) production; this process is orchestrated by HIF-1α through the induction of glycolytic genes [78]. Interestingly, while MYC and HIF-1α broadly activate glycolytic enzymes, PGAMs remain an exception and are exclusively regulated by RB1. This unique transcriptional control by RB1 proposes a specialized role for PGAMs in cancer metabolism distinct from other glycolytic enzymes. The frequent inactivation of RB1 during the tumor progression phase further complicates the landscape of how altered tumor metabolism contributes to the gain of malignant phenotypes. While RB1 loss promotes tumorigenesis by enhancing cellular proliferation and survival, it simultaneously induces metabolic vulnerabilities such as increased reactive oxygen species (ROS) production; such ambivalent cellular state must be counterbalanced through enhanced glutaminolysis and lipid degradation [6, 9]. Our findings that RB1 loss shifts the primary carbon source from glucose to glutamine via PGAM downregulation in 53KOLS cells highlight thus far an unknown mode of metabolic adaptation that supports tumor growth under metabolic stress. This adaptation appears to optimize the use of available nutrients by rechanneling carbons from glutathione synthesis into the TCA cycle to sustain energy production and anabolic processes.

In conclusion, the role of RB1 in transcriptional control of PGAMs underscores its importance in remodulating metabolic pathways in both cancer and normal tissue differentiation extending beyond its canonical functions in cell cycle control and differentiation. These insights into the metabolic functions of RB1 may open new avenues for therapeutic intervention by targeting metabolic alterations associated with RB1 dysfunction.

## Materials and methods

### Cell lines and primary cell culture

Primary 53KOLS cells were prepared as described previously [9] from *Trp53*-knockout mice and maintained in α-modified Eagle’s medium (αMEM, 041-29775, Fujifilm Wako Pure Chemical Co., Tokyo, Japan) supplemented with 10% fetal bovine serum (FBS, A5256701, (Thermo Fisher Scientific, Waltham, MA, USA). SNU-601, SNU-638, SNU-738, and AGS (gifted from Dr. Masanobu Oshima, Kanazawa University) cells were maintained in RPMI (189-02025, Fujifilm Wako Pure Chemical Co.) supplemented with 10%FBS. C2C12 (RCB0987, RIKEN BRC, Tsukuba, Japan) cells were maintained in high glucose DMEM (043-30085, Fujifilm Wako Pure Chemical Co.) supplemented with 20% FBS. 3T3L1 (IFO50416, JCRB, Osaka, Japan) and HEK293T (RCB2202, RIKEN BRC) cells were maintained in high glucose DMEM supplemented with 10% FBS. For inducing differentiation in C2C12 cells, 2 × 105 cells were plated onto 6-well plate. After 24 h incubation, the medium was changed to low glucose DMEM (041-29775, Fujifilm Wako Pure Chemical Co.) supplemented with 2% horse serum (16050130, Thermo Fisher Scientific). Medium was replaced every 2 days up to 7 days. For inducing differentiation in 3T3L1, cells were plated onto 6-well plate at 3 × 105 density. After 48 h (day 0), cells reached confluence then medium was replaced to fresh medium every 24 h. On day 2, cell differentiation was induced by treatment with DMEM containing 10% FBS, 0.5 mM 3-isobutyl-1-methylxanthine (19624-44, Nacalai Tesque, Kyoto, Japan), 0.25 μM dexamethasone (D2915, Merck Millipore, Billerica, MA, USA) and 1 μg/ml insulin (I0516, Merck Millipore) for 48 h. On day 4, the medium was changed to DMEM containing 10% FBS, 1 μg/ml insulin. This medium was refreshed every 48 h up to day 11. For control cells, medium was replaced with fresh media every 48 h.

### Mouse studies

Trp53 knockout mice [9] were obtained from RIKEN BRC (CDB0001K). C57BL/6 mice were purchased from Japan SLC.

### Metabolome analysis

One and half million cells were washed with 5% mannitol (139-00847, Fujifilm Wako Pure Chemical Co.) solution and treated with 800 μl of methanol (138-14521, Fujifilm Wako Pure Chemical Co.) for 30 s. The resultant cell lysates were treated with 550 μl of Milli-Q water containing 10 μM of internal standard (H3304-1002, Human Metabolome Technologies, Tsuruoka, Japan) for 30 s and centrifuged at 2300 × *g* and 4 °C for 5 min. The supernatants were filtered through a Millipore 5-kDa cutoff filter by centrifugation at 9100 × *g* and 4 °C for 120 min. The filtrated solution was evaporated for concentration and re-suspended in 50 μl of Milli-Q water for CE-MS analysis. Data was normalized and analyzed by Metaboanalyst 6.0 (www.metaboanalyst.ca/) [79].

### Immunoblot analysis

Immunoblotting of whole cell lysates was performed as described previously [9]. Primary antibodies were as follows: Total RB (sc-50, Santa Cruz, Dallas, TX, USA), PGAM2 (PA5-24006, Thermo Fisher Scientific), PGAM1 (NBP1-49532, Novus Biologicals, Abingdon, United Kingdom), PKM1 (#7067, Cell Signaling Technology, Danvers, MA, USA), PKM2 (#4053 Cell Signaling Technology), HK1 (#2024, Cell Signaling Technology), HK2 (#2867, Cell Signaling Technology), PFKP (13389-1-AP, Proteintech, Rosemont, IL, USA), PFKM (55028-1-AP, Proteintech), PKM1 (#7607, Cell Signaling Technology), PKM2 (#4053, Cell Signaling Technology), ENO3 (55234-1-AP, Proteintech), MPC1 (#14462, Cell Signaling Technology) MPC2 (20049-1-AP, Proteintech), Phospho-PDH (AP1062, Merck Millipore), whole PDH (#3205, Cell Signaling Technology), LDHA (#2012, Cell Signaling Technology), α-Tubulin (#CP06, Merck Millipore), MEF2A (#9736, Cell Signaling Technology), MEF2D (610775, BD Biosciences, San Jose, CA), KDM5A (18825-1-AP, Proteintech), HRP-linked anti-rabbit antibody (#7074, Cell Signaling Technology) and HRP-linked anti-mouse antibody (#7076, Cell Signaling Technology).

### Glucose uptake assay

53KOLS, AGS and SNU-638 cells were plated onto 6-well-type plate at 1–3 × 105 cells per well. After 24 h culture, medium was replaced with fresh complete culture medium containing 50 μM 2-deoxy-2-[(7-nitro-2,1,3-benzoxadiazol-4-yl)amino]-D-glucose (2-NBDG) (23002-v, Peptide Institute, Osaka, Japan), and incubated at 37 °C for 30 min. The cells were then washed with PBS twice, trypsinized, and suspended in 500 μl of PBS containing 5% FBS. 30,000 cells fluorescing by 2-NBDG were analyzed by FACSCanto (BD Biosciences) according to the manufacturer’s instructions.

### Generation of lentivirus

MISSION TRC shRNA target sets for mouse Rb (TRCN0000042543 and TRCN0000042544), human RB1 (TRCN0000040163 and TRCN0000010419), mouse Mef2a (TRCN0000333987 and TRCN0000333988), mouse Mef2d (TRCN0000085270 and TRCN0000085271), human KDM5A (TRCN0000014629 and TRCN0000014630), and negative control (Scramble; SHC002) were purchased from Merck Millipore. Generation and infection of lentivirus were performed according to the manufacturer’s instruction. pLenti6.3-hPGAM1 was constructed by gateway system. For lentivirus production, 3 × 106 HEK293T cells were transfected with 5 μg of pLenti6.3 expression vector or plko.1 shRNA expression vector together with 4.5 μg of psPAX2, 0.5 μg of pCMV-VSVG, and 30 μg polyethyleneimine (24765, Polyscience, Inc., Warrington, PA) in 1 ml of OPTI-MEM (31985-070, Thermo Fisher Scientific). After 72 h transfection, the media containing lentivirus particles were collected, removed debris by filtration, and concentrated using solution of polyethylene glycol for overnight.

### Generation of retrovirus

Retroviruses were harvested from supernatant of Platinum-E (VPK-300, Cell Bio Labs, Inc., San Diego, CA, USA) retroviral packaging cell line purchased from Cell Bio Lab. Platinum-E cells were maintained in DMEM supplemented with 10% FBS and transfected with pMXs or pMXs- mouse Pgam2. For retrovirus production, 4 × 106 Platinum-E cells were transfected with 10 μg of each pMXs vector and 30 μg polyethyleneimine in 1 ml of OPTI-MEM. After 72 h transfection, supernatants were collected, removed debris by filtration, and concentrated using a polyethylene glycol solution for overnight.

### RT-qPCR

Total RNA was isolated from cultured cells using TRIzol (15596018, Thermo Fisher Scientific) according to the manufacturer’s instructions. Quantitative PCR of total RNA was carried out as described previously using high-capacity cDNA synthesis kit (4387406, Thermo Fisher Scientific) and Taqman probes. Taqman probes used are as follow: mouse *Actb* (Mm00607939_s1), mouse *Rb* (Mm00485586_m1), mouse *Hprt* (Mm00446968_m1), mouse *Nanog* (Mm02019550_s1), mouse *Pgam2* (Mm01187768_m1), mouse *Myh7* (Mm00600555_m1), mouse *Myog* (Mm00446194_m1), mouse *Myod1* (Mm00440387_m1) human *ACTB* (Hs99999903_m1), human *RB* (Hs01078066_m1), human *PGAM1* (Hs01652468_g1) and human *HPRT* (Hs02800695_m1). The relative level of gene expression was normalized using the level of *Actb* or *Hprt*. The values represent the average of three biological replicates.

### Cap analysis gene expression (CAGE) sequencing

2 × 10^6^ cells were plated onto D100 dish and cultured with complete medium. After 24 h, total RNA was collected and purified by RNeasy Mini Kit (#74106, Qiagen). CAGE library preparation, sequencing, mapping, and following gene expression and motif discovery analysis were performed by DNAFORM (Yokohama, Japan). In brief, the RNA quality was assessed by Bioanalyzer (Technologies, Santa Clara, CA, USA) to ensure that RNA integrity number is over 7.0　and that A260/280 and 260/230 ratios are over 1.7. First strand cDNAs were transcribed to the 5′ end of capped RNAs, attached to CAGE “bar code” tags, and the sequenced CAGE tags were mapped to the human hg19 genomes using BWA software (v0.5.9) after discarding ribosomal or non-A/C/G/T base-containing RNAs. For tag clustering, CAGE-tag 5′ coordinates were input for RECLU (RIKEN clustering), with a maximum irreproducible discovery rate of (IDR) 0.1 and minimum tags per million (TPM) value of 0.1. Native elongating transcript–cap analysis of gene expression (NET-CAGE) was performed as described in the study [40]. The raw data of CAGE is available in DNA Data Bank of Japan (DRA018902).

### Extracellular flux analysis

2.0–4.5 × 106 cells were plated onto XF24 cell culture plate with complete medium. After 12 h culture, medium was changed with unbuffered DMEM (D5030, Merck Millipore). For measuring OCR and ECAR after glucose stimulation, cells were incubated in unbuffered DMEM supplemented with 2 mM glutamine at 37 °C for 1 h without CO_2_ control, then glucose (10.5 mM, 041-00595, Fujifilm Wako Pure Chemical Co.) and 2-deoxy glucose (100 mM, D0051, Tokyo Chemical Industry Co., Tokyo, Japan) were sequentially injected to stimulate the cells in culture plate. For measuring OCR and ECAR after glutamine (4 mM, 074-00522, Fujifilm Wako Pure Chemical Co.) or pyruvate (4 mM, 191-03061, Fujifilm Wako Pure Chemical Co.) stimulation, cells were incubated in unbuffered DMEM supplemented with 0.1% BSA at 37°C for 1 h without CO_2_ control, then glutamine (4 mM) or pyruvate (1 mM) was injected to stimulate the cells in culture plate. For mitochondrial stress test, cells were incubated in unbuffered DMEM supplemented with 25 mM glucose, 2 mM glutamine and 1 mM pyruvate at 37 °C for 1 h without CO_2_ control, then Oligomycin A (1 μM, #9906, Cell Signaling Technology) was injected to inhibit ATPase V. Maximal OCR was induced by exposing cells to mitochondrial uncoupler FCCP (300 nM for 53KOLS, 1 μM for AGS, C2920, Merck Millipore). Rotenone and Antimycin A (1 μM each, R8875 and A8674, Merck Millipore) were added to disrupt all mitochondria-dependent respiration. OCR and ECAR measurements were taken for a 3-minute period, followed by 2 min mixing and 3 min incubation. Three basal rate measurements were taken before injection of nutrients to stimulate metabolism or mitochondrial manipulating reagents. OCR and ECAR were normalized by cell number.

### Glutamine uptake assay

3 × 105 53KOLS cells were plated onto 6-well plate in DMEM supplemented with 10% FBS. After 24 h of culture, medium was changed to DMEM supplemented with 10% FBS and ^14^C-labeled glutamine (0.5 μCi/mL, MC-1124, Moravek Biochemicals Inc., Brea, CA, USA). After incubation for 5 min, cells were washed with cold PBS twice, then lysed in 200 μl of 0.2% SDS/0.2 M NaOH. Cell lysates were neutralized by HCl and mixed with 4 ml of Hionic-Fluor scintillation cocktail (#6013311, Revvity Inc., Waltham, MA, USA), and ^14^C activity was measured using liquid scintillation counter (LSC-5200, Aloka Co., Tokyo, Japan).

### ^14^C carbon tracing analysis

2 × 105 53KOLS cells were plated onto 6-well plate in DMEM supplemented with 10% FBS. After 24 h culture, medium was changed to DMEM supplemented with 10% dialyzed FBS and ^14^C labeled carbon sources or tritium water, and cells were incubated at 37 °C for 24 h. The tracers were used as following: [U-^14^C]-glucose (6.25 μCi/ml, MC-144W, Moravek Biochemicals Inc.), [U-^14^C]-glutamine (62.5 μCi/ml), and ^3^H_2_O (20 v/v%, ART0194A, American Radiolabeled Chemicals, St. Louis, MO, USA). Then cells were washed with cold PBS twice and treated with 500 μl of 60% hexane/40% isopropanol to extract lipids. The Organic solvents were linsed twice with 300 μl of PBS, and non-polar phase was collected. The extracted fraction was dried, dissolved in 200 μl of chloroform, and mixed with 4 ml of Hionic-Fluor scintillation cocktail. Quantified c.p.m. by liquid scintillation counting was normalized by cell number.

### Sphere formation assay

Sphere formation assay was carried out as previously described [30]. In Brief, cells under monolayer culture were detached from dish and dissociated through a 40 μm cell strainer and then cultured in αMEM supplemented with B27 (17504044, Thermo Fisher Scientific), human bFGF (068-04544, Fujifilm Wako Pure Chemical Co.), human EGF (335-44981, Fujifilm Wako Pure Chemical Co.), 1% methylcellulose (132-05055, Fujifilm Wako Pure Chemical Co.) without serum at a density of 5 × 103 on 24-well ultra-low attachment plate. After 14 days culture, spheres were observed with the assistance of an inverted phase contrast microscopy and analyzed by BZ analysis software on BZ-9000 (Keyence, Osaka, Japan) using hybrid cell counting module. Sphere-forming units were determined at a given day by counting cell aggregates with larger than 3000 (53KOLS) or 5000 (AGS cells) μm2 surface area and with the ratio of the longest diameter and the shortest diameter (L/S ratio) less than 2. The whole area in a dish was scanned by automated microscope, and sphere number per dish was calculated from tiled image data.

### Data analysis of RNA sequence and DNA microarray

The raw data of RNA sequence in 53KOLS were obtained from DNA Data Bank of Japan (DRA002910). The raw data of microarray analysis were obtained from Gene Expression Omnibus database (GSE43145, GSE11425, and GSE3014). TCGA dataset (Gastric cancer) was obtained from cBioportal (https://www.cbioportal.org/).

### mtDNA quantification

Genomic DNAs were extracted from 5 × 105 cells using pure link Genomic DNA Mini Kit (K1820-01, Thermo Fisher Scientific). The relative mtDNA copy number to nDNA was determined using quantitative PCR with Fast SYBR Green Master Mix (#4385614, Thermo Fisher Scientific). Forward and reverse primers were as following: mouse hexokinase 2 gene intron9 (5’-GCCAGCCTCTCCTGATTTTAGTGT-3’ and GGGAACACAAAAGACCTCTTCTGG-3’), mouse 16S rRNA (5’-CCGCAAGGGAAAGATGAAAGAC-3’ and 5’-TCGTTTGGTTTCGGGGTTTC-3’), human β-globin (5′- GAAGAGCCAAGGACAGGTAC-3′ and 5′- CAACTTCATCCACGTTCACC-3′) and human ND1 (5′-AACATACCCATGGCCAACCT-3′ and 5′- AGCGAAGGGTTGTAGTAGCCC-3′).

### Flow cytometry

Mitochondrial membrane potential was measured with tetramethyl rhodamine methyl ester (TMRM, T668, Thermo Fisher Scientific). Mitochondrial mass was measured with MitoTracker green (M7514, Thermo Fisher Scientific). 3 × 10^5^ cells were incubated with MitoTracker green or TMRM at 37 °C for 30 min in DMEM supplemented 10% FBS. 30,000 cells suspended in 500 μl of PBS containing 5% FBS were analyzed by FACSCanto (BD Biosciences).

### ChIP-qPCR

Primary antibodies were as follows: H3K4me2 (MABI0303, Medical Biological Laboratories Co., Tokyo, Japan), H3K9Ac (MABI0305, Medical Biological Laboratories Co.), V5 (ab15828, Abcam, Cambridge, United Kingdom), and mouse IgG (18413, Abcam). 3 × 10^6^ cells were fixed with 1% formaldehyde (064-00401, Fujifilm Wako Pure Chemical Co.) for 10 min at room temperature. Crosslinking was quenched by adding glycine to a final concentration of 0.125 M. Cells were washed with ice-cold PBS and harvested in PBS. The nuclear fraction was extracted by first resuspending the pellet in 1 ml of NP40 buffer (50 mM Tris-HCl (pH 8.0), 10 mM NaCl, 0.5% NP-40) for 10 min at 4 °C. Cells were then pelleted and resuspended in 1 ml of shearing buffer (50 mM Tris-HCl (pH 8.0), 1 mM EDTA, 0.1% SDS, 1% Triton X-100, 0.1% deoxycholate, and 167 mM NaCl) and sonicated by sonicator (Q125, Qsonica LLC., Newtown, CT, USA). Lysate was centrifuged for 15 min at 14,000 rpm to remove the debris. The sample was then incubated with 20 μl of Dynabeads anti mouse IgG (11201D, Thermo Fisher Scientific) and primary antibody for overnight at 4 °C. The beads were then washed in low salt RIPA buffer (50 mM Tris-HCl pH 8.0, 150 mM NaCl, 1 mM EDTA, 1% Triton X-100, 0.1% deoxycholate, and 0.1% SDS) for 5 min at 4 °C, high salt RIPA buffer (50 mM Tris-HCl pH 8.0, 500 m NaCl, 1 mM EDTA, 1% Triton X-100, 0.1% deoxycholate, and 0.1% SDS) for 5 min at 4 °C and LiCl wash buffer (10 mM Tris-HCl pH 8.0, 1 mM EDTA, 0.5% Triton X-100, and 0.5% deoxycholate) for 5 min at 4°C. DNA was eluted and de-cross-linked in elution buffer (10 mM Tris-HCl, 300 mM NaCl, 5 mM EDTA, and 0.5% SDS) for overnight at 65 °C. RNA and protein were digested with 0.5 mg/ml RNase A for 30 min at 37 °C followed by 0.5 mg/ml Proteinase K for 1 h at 55 °C. NA was purified with QIAquick PCR Purification Kit (28104, Qiagen, Valencia, CA, USA). Forward and reverse primers were as following; mouse Pgam2 (5’-AGGCAACCAGTAGCAGAACTG-3’, 5-‘TATGGGGCTTGTCATCTGAGC-3’) and PGAM1 (5’-TCTCATTTGAATGACTGGCAGAA-3’, 5’-GGCCCTCTTAGAAATTCAGCA-3’), and Human Negative control: (71001, Active motif, Carlsbad, CA, USA).

## Quantitation and statistical analysis

### Statistical analysis

Statistical significance was detected by using unpaired Student’s *t* test between two groups or one-way ANOVA followed by post hoc Tukey’s test among more than three groups. CAGE analysis was performed using DESeq2, *p*-values less than 0.05 were considered statistically significant.

## Supplementary information

Supplemental Figures

Uncropped Immunoblotting

## Data Availability

The CAGE and NET-CAGE analyzed during the current study are available from DNA Data Bank of Japan (DRA018902). The datasets used in the current study were published previously. All genetic materials used for this paper are available from the authors on request.
