# Supplementary material for: RB1 controls differentiation through positive regulation of phosphoglycerate mutases
Source: Cell Death Dis. 2025 Jul 24;16(1):559. doi: 10.1038/s41419-025-07850-3 (PMC12290115; doi:10.1038/s41419-025-07850-3)
Supplement: Supplementary file 1 — Supplemental Figures [file 41419_2025_7850_MOESM1_ESM.pdf]

SUPPLEMENTARY FIGURES

RB1 controls differentiation through positive regulation of phosphoglycerate mutases

Susumu Kohno[1,*], Nobuyuki Okahashi[2], Yuansong Wan[1,3], Hai Yu[1], Yujiro Takegami[4], Paing Linn[1,5], Naoko Nagatani[1], Shunsuke Kitajima[1,6], Teruo Kawada[7], Fumio Matsuda[2], Hiroshi Shimizu[2] and Chiaki Takahashi[1,*]

Supplementary Figures

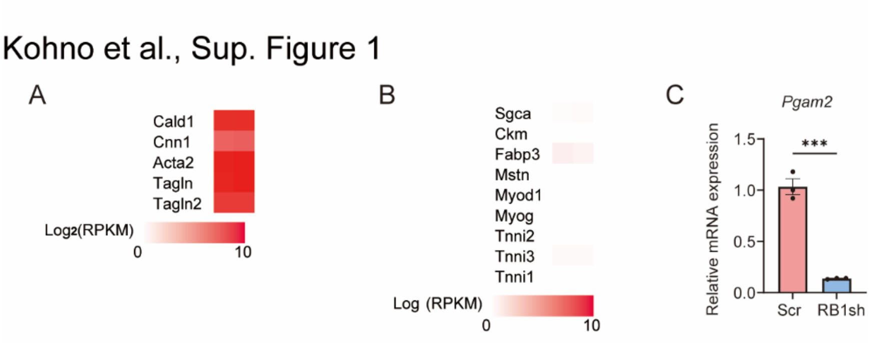

Supplementary Fig. 1

Expression of myogenic genes in 53KOLS cells

(a) Expression of smooth muscle related genes in 53KOLS cells. (b) Expression of skeletal muscle related genes in 53KOLS cells. (c) mRNA expression of Rb1 in 53KOLS cells transduced with shRNA targeting mouse Rb.

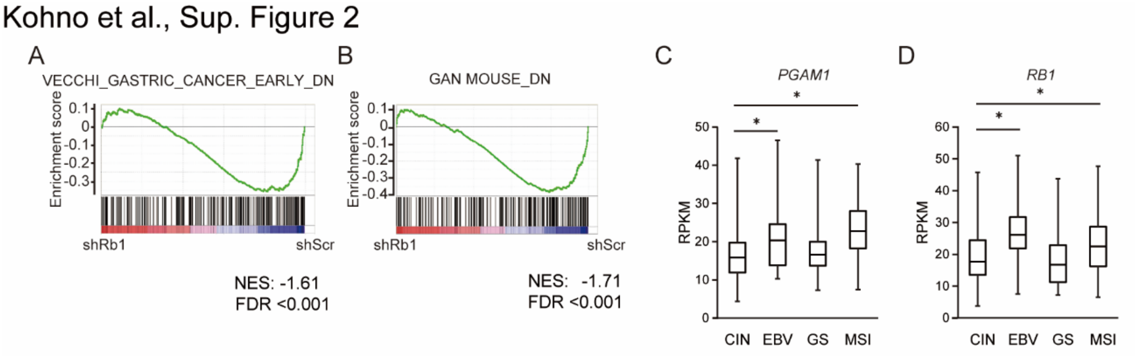

Supplementary Fig. 2

The correlation between Rb loss signature in 53KOLS cells and feature of gastric

cancer

(a) GSEA result of h.C2.all.v2023.2.Hs.symbols.gene.sets comparing gene signature in human gastric cancer cells to genes differentially expressed in 53KOLS cells transduced with shRNA targeting Rb versus scrambled sequence. (b) GSEA result of gene signature in downregulated genes in Gan mouse comparing gene signature in gastric cancer cells developed in Gan mouse to genes differentially expressed in 53KOLS cells transduced with shRNA targeting Rb versus scrambled sequence. (c, d) Box plot of mRNA expression levels of PGAM1 and RB1 in four distinct gastric cancer subtypes. Tukey's HSD test was performed. * $P < 0.05$.

Kohno et al., Sup. Figure 3

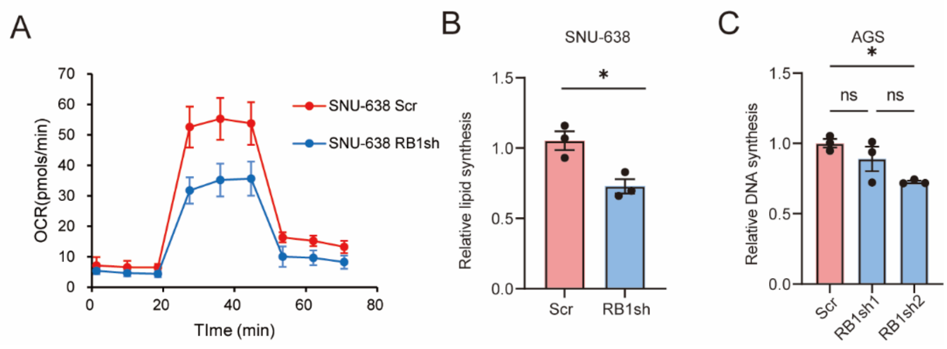

Supplementary Fig. 3

RB1 depletion in SNU-638 cells reduces utilization of carbon source derived from glucose

(a) ECAR was measured in SNU-638 cells transduced with shRNA targeting RB1 or scrambled sequence. Cells were sequentially treated with 10.5 mM glucose and then 100 mM 2-deoxy glucose (2-DG). (b) Relative 2-NBDG fluorescence intensity, which is equivalent to glucose uptake, in SNU-638 cells transduced with shRNA targeting RB1 or scrambled sequence. (c) Relative incorporation of [U-^{14}C]-glucose into nuclear DNA. AGS cells were cultured with [U-^{14}C]-glucose (6.25 μCi/ml) for 24 hrs. ^{14}C activity was measured using liquid scintillation counter. Tukey's HSD test was performed. * $P < 0.05$.

Kohno et al., Sup.Figure 4

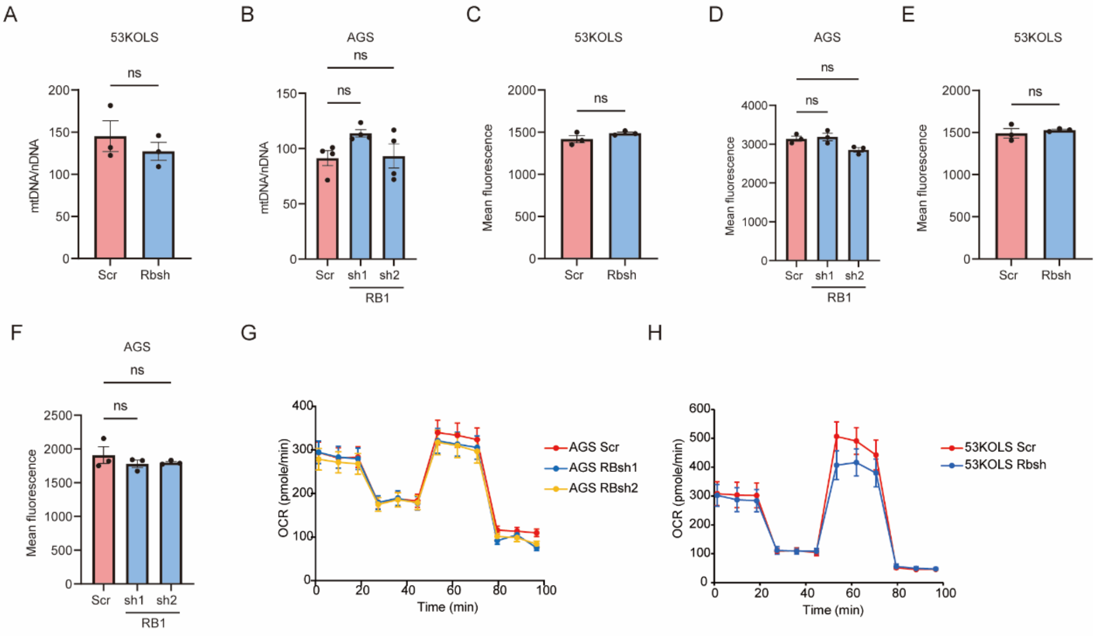

Supplementary Fig. 4

RB1 depletion does not affect mitochondrial function

(a) Relative copy number of mitochondrial DNA versus nuclear DNA in 53KOLS cells. 16S rRNA was measured as a representative of mitochondrial DNA and normalized to nuclear DNA hexokinase 2. (b) Relative copy number of mitochondrial DNA versus nuclear DNA in AGS cells. NADH-ubiquinone oxidoreductase chain 1 (ND1) was measured as a representative of mitochondrial DNA and normalized to nuclear DNA β-globin. (c) Mitochondrial mass in 53KOLS cells transduced with the indicated shRNA was measured by staining with mitotracker green. (d) Mitochondrial mass in AGS cells transduced with the indicated shRNA was measured by staining with mitotracker green. (e) Mitochondrial membrane potential in 53KOLS cells transduced with the indicated shRNA was measured by staining with tetramethylrhodamine ester (TMRM) on flowcytometer. (f) Mitochondrial membrane potential in AGS cells transduced with the indicated shRNA was measured by staining with TMRM on flowcytometry. (g) Mitochondrial stress test in AGS cells transduced with RB1 shRNA. Cells were subsequently stimulated with oligomycin, carbonyl cyanide 4-(trifluoromethoxy)phenylhydrazone (FCCP) and rotenone/antimycin A. (h) Mitchondrial stress test in 53KOLS cells transduced with Rb sh RNA. Cells were subsequently stimulated with oligomycin, FCCP and rotenone/antimycin A.

Kohno et al., Sup. Figure 5

A

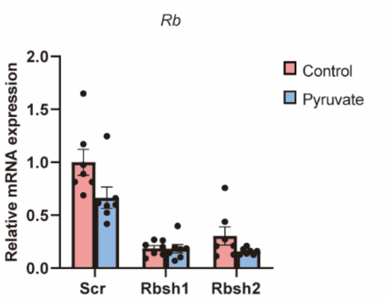

Supplementary Fig. 5

Rb expression level in Rb-depleted C2C12 cells treated with pyruvate

(a) Relative mRNA expression of Rb in C2C12 cells transduced with shRNA targeting Rb or scrambled sequence.
