# Supplementary material for: RB1 controls differentiation through positive regulation of phosphoglycerate mutases
Source: Cell Death Dis. 2025 Jul 24;16(1):559. doi: 10.1038/s41419-025-07850-3 (PMC12290115; doi:10.1038/s41419-025-07850-3)

SUPPLEMENTARY DATA: UNCROPPED IMMUNOBLOIING

RB1 controls differentiation through positive regulation of phosphoglycerate mutases

Susumu Kohno[1,*], Nobuyuki Okahashi[2], Yuansong Wan[1,3], Hai Yu[1], Yujiro Takegami[4], Paing Linn[1,5], Naoko Nagatani[1], Shunsuke Kitajima[1,6], Teruo Kawada[7], Fumio Matsuda[2], Hiroshi Shimizu[2] and Chiaki Takahashi[1,*]

Figure 1G Rb

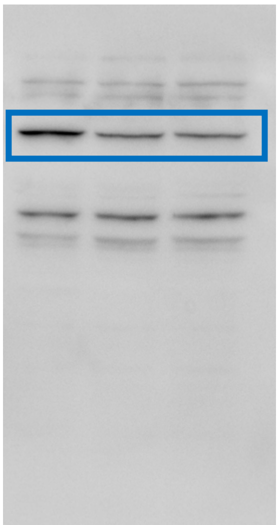

Figure 1G Pgam2

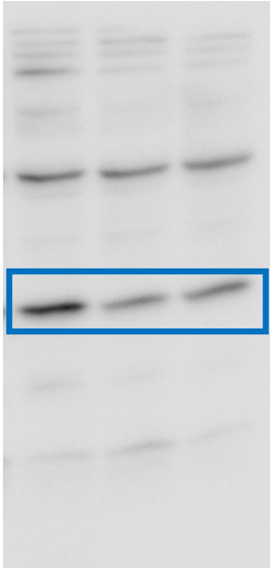

Figure 1G Pgam1

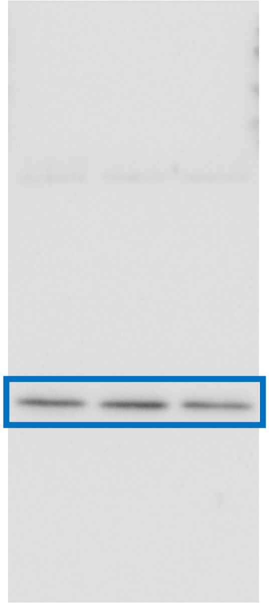

Figure 1G Hk1

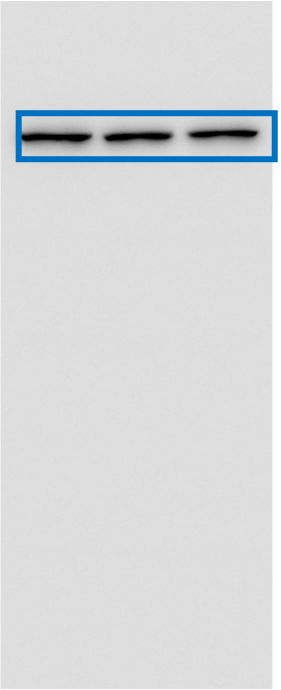

Figure 1G Hk2

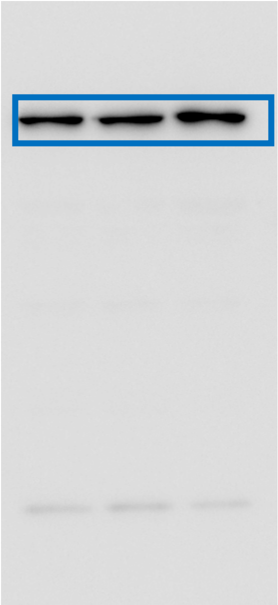

Figure 1G Pfkp

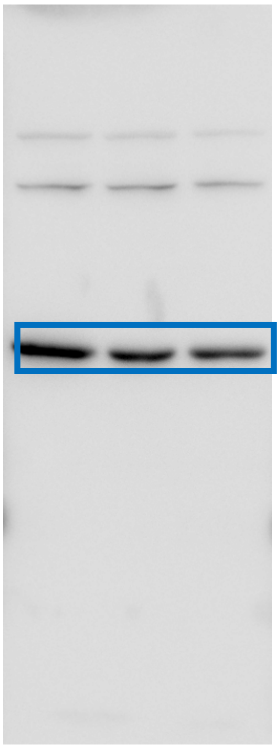

Figure 1G Pfkm

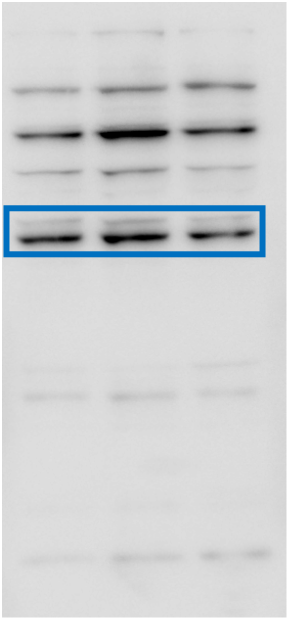

Figure 1G Pkm1

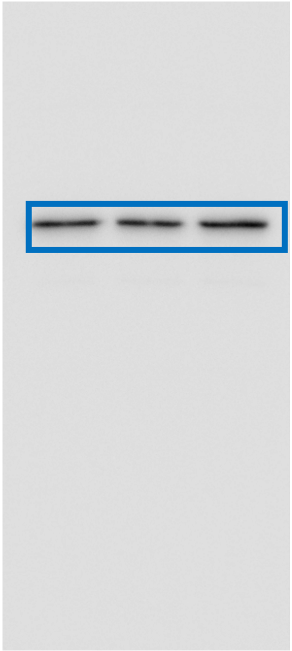

Figure 1G Pkm2

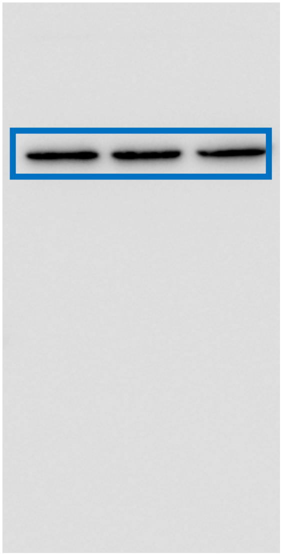

Figure 1G Eno3

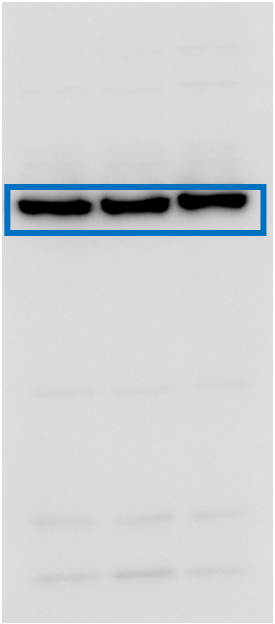

Figure 1G Mpc1

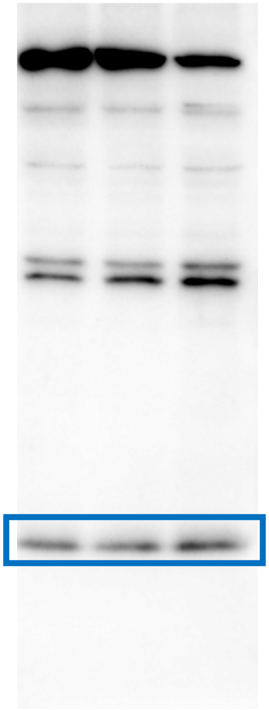

Figure 1G Mpc2

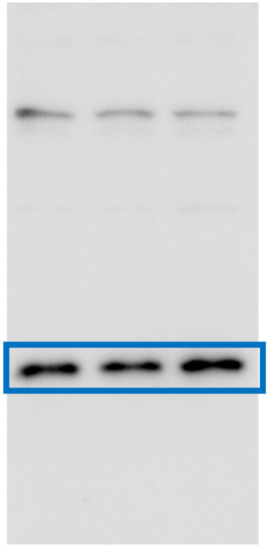

Figure 1G pPdh

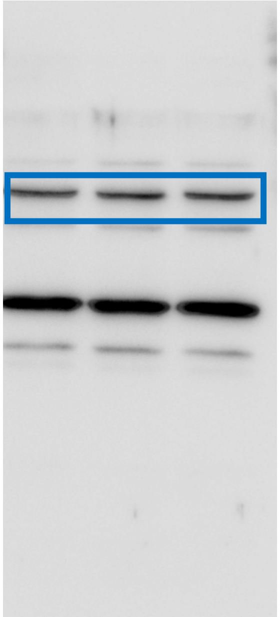

Figure 1G Pdh

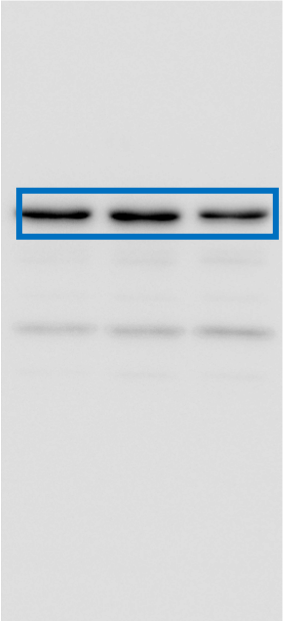

Figure 1G α-tubulin

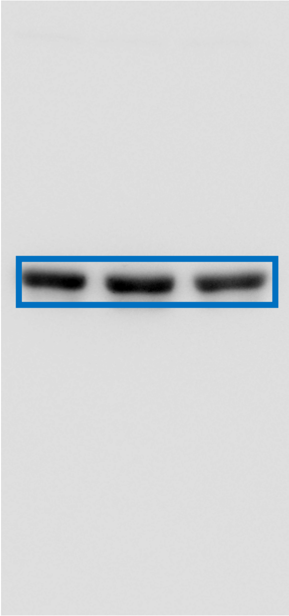

Figure 1N SNU-638
RB

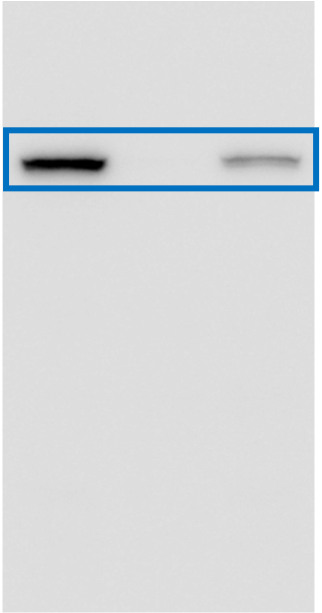

Figure 1N SNU-638
PGAM1

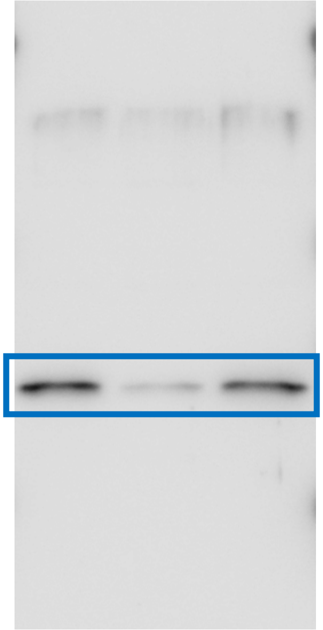

Figure 1N SNU-638
HK1

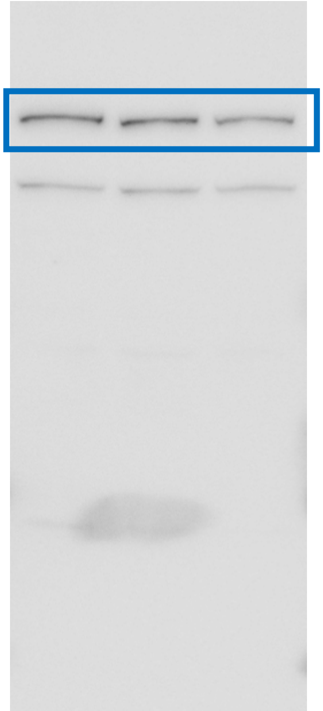

Figure 1N SNU-638

HK2

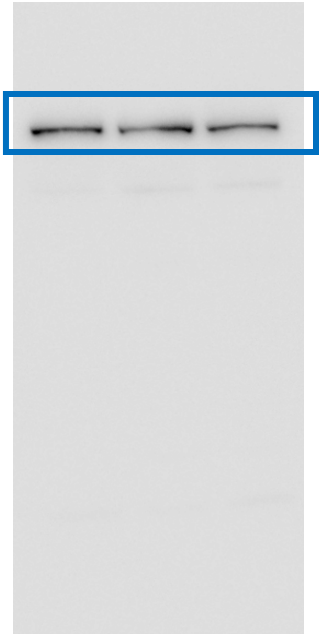

Figure 1N SNU-638

PFKP

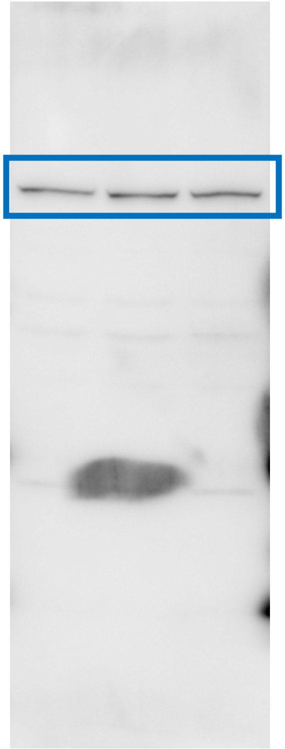

Figure 1N SNU-638

PKM1

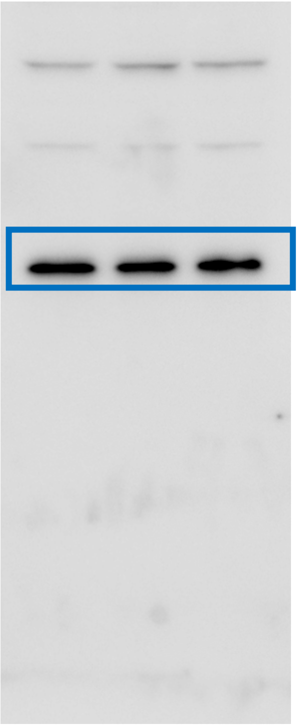

Figure 1N SNU-638

PKM2

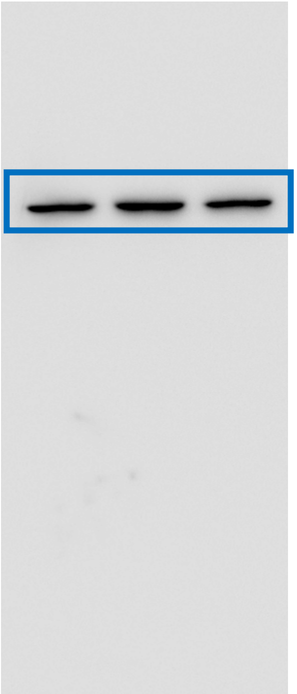

Figure 1N SNU-638

MPC1

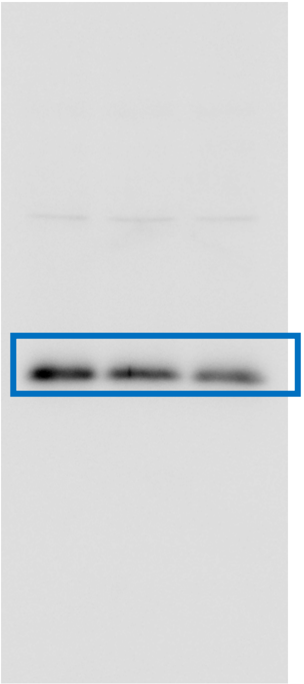

Figure 1N SNU-638

MPC2

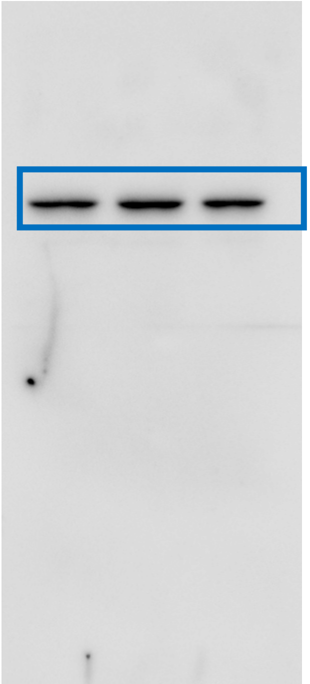

Figure 1N SNU-638
pPDH

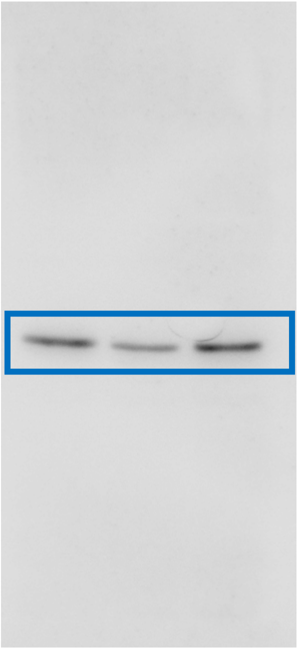

Figure 1N SNU-638
PDH

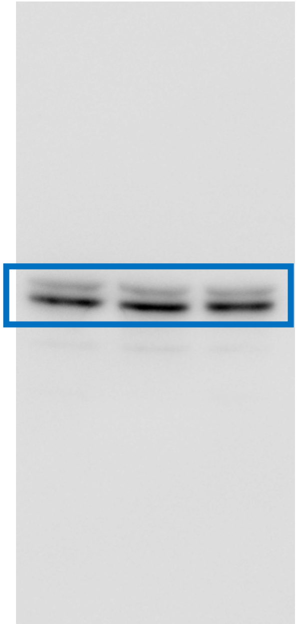

Figure 1N SNU-638
LDHA

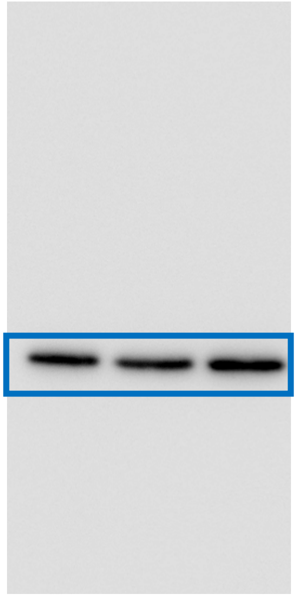

Figure 1N SNU-638
α-tubulin

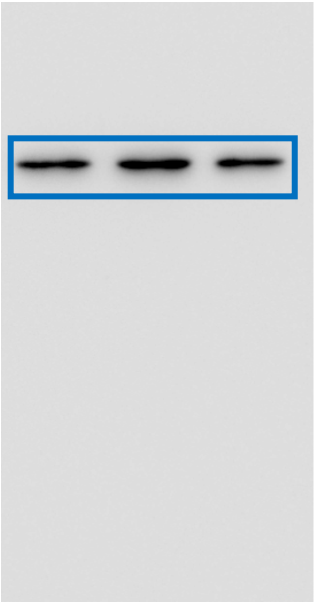

Figure 1N AGS
RB

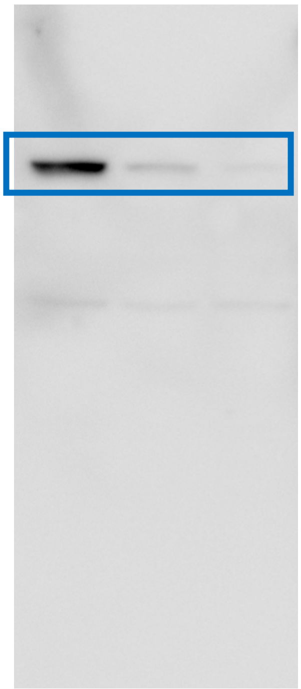

Figure 1N AGS
PGAM1

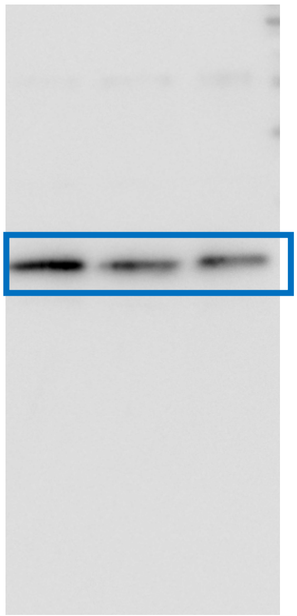

Figure 1N AGS
HK1

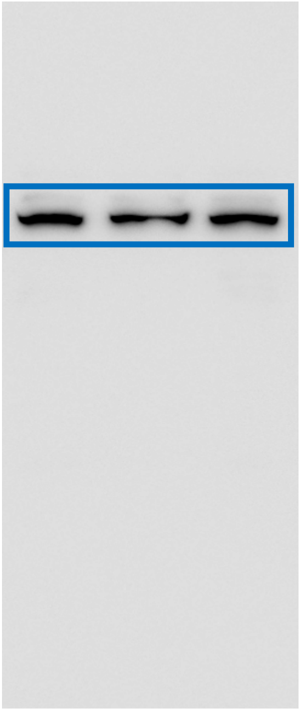

Figure 1N AGS
HK2

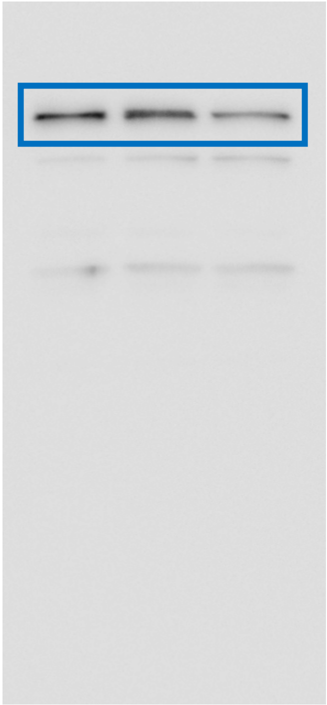

Figure 1N AGS
PFKP

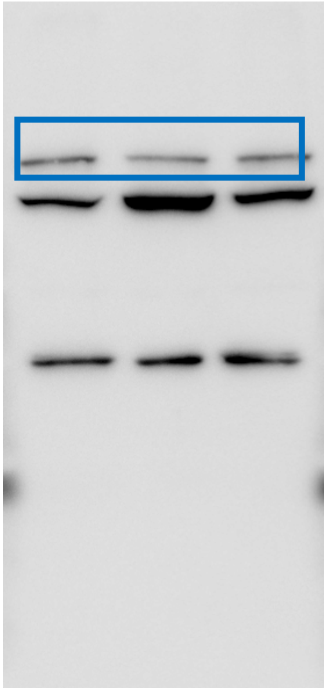

Figure 1N AGS
PKM1

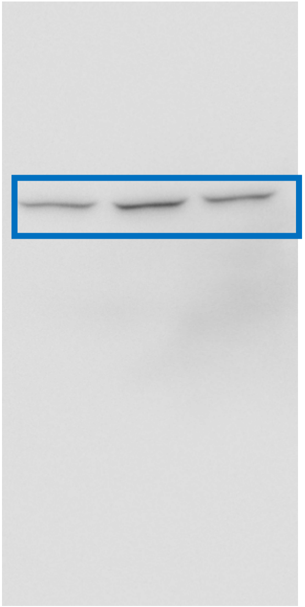

Figure 1N AGS
PKM2

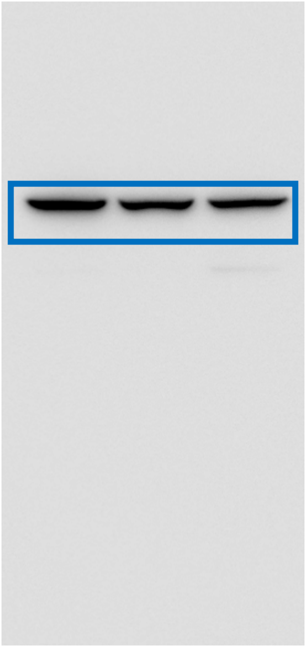

Figure 1N AGS
MPC1

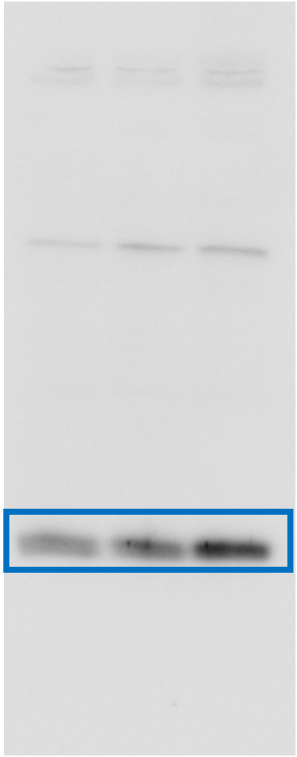

Figure 1N AGS
MPC2

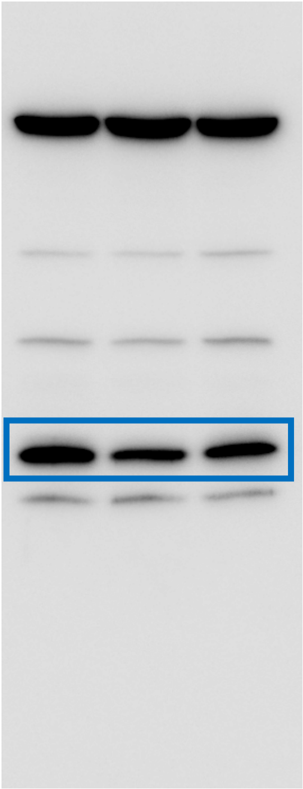

Figure 1N AGS
pPDH

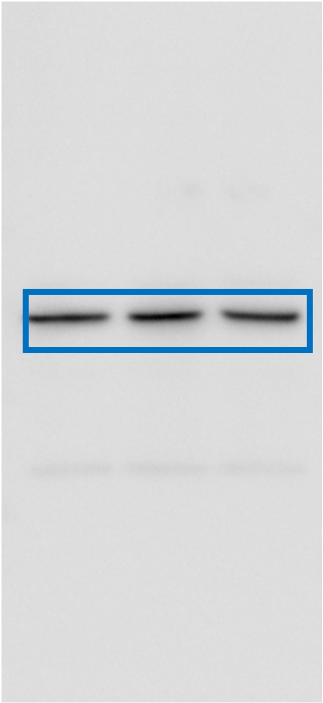

Figure 1N AGS
PDH

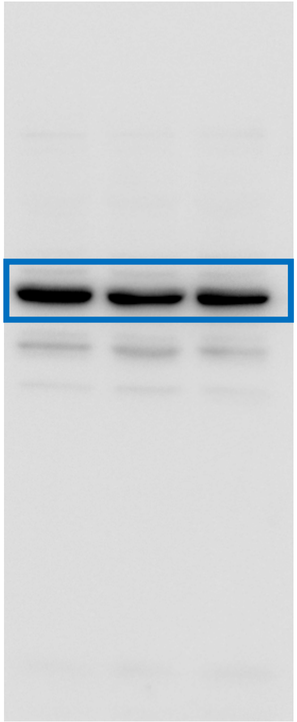

Figure 1N AGS
LDHA

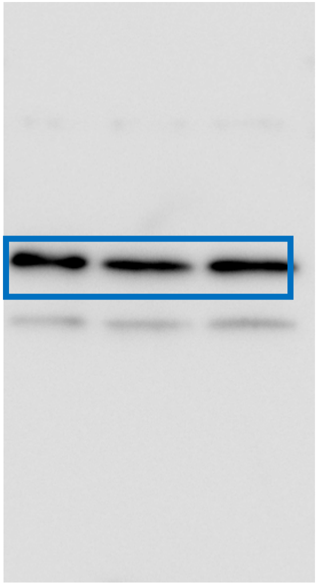

Figure 1N AGS
α-tubulin

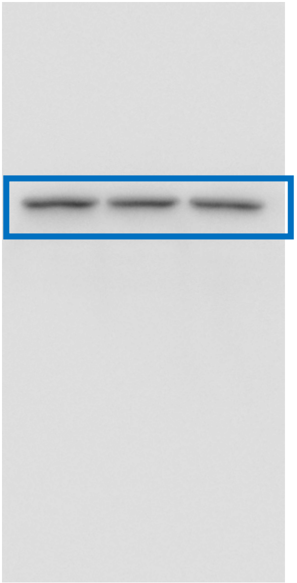

Figure 2J Pgam2

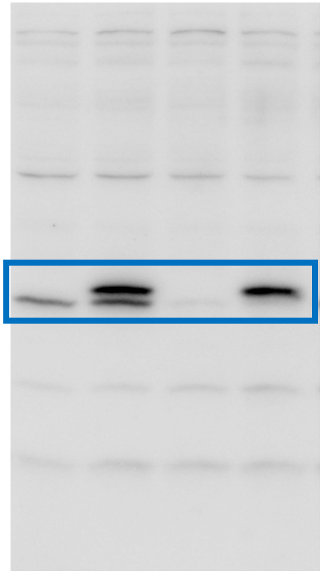

Figure 2J Pgam1

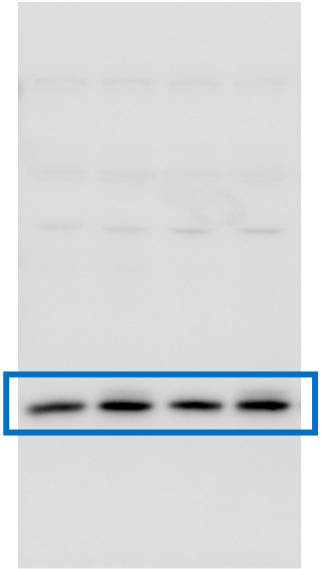

Figure 2J α-tubulin

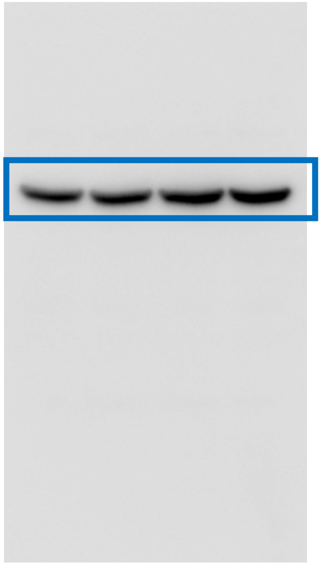

Figure 4B Mhc

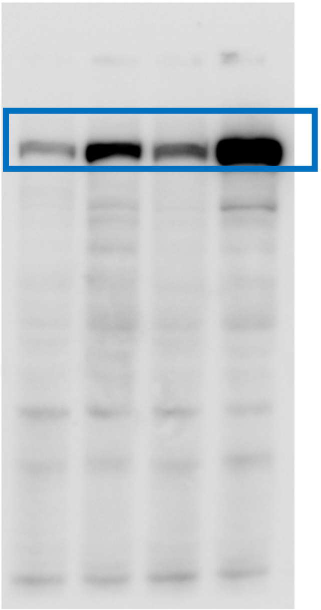

Figure 4B Pgam2

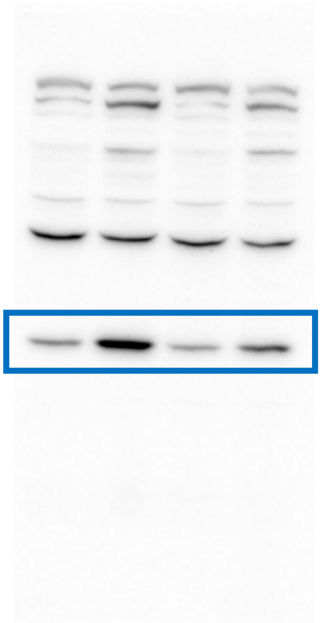

Figure 4B Phospho-Rb

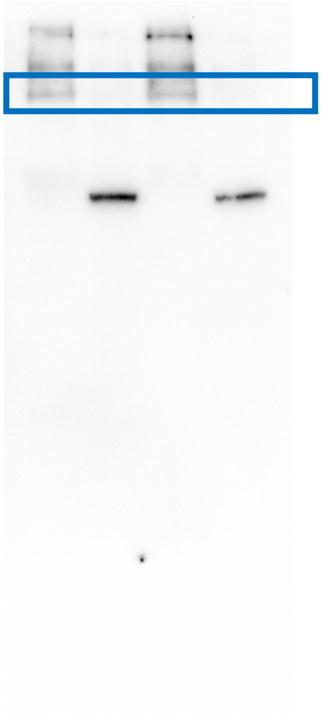

Figure 4B Rb

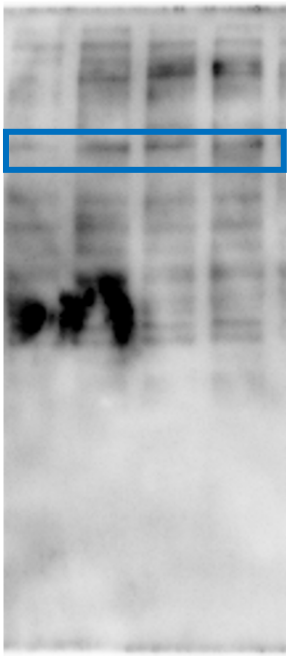

Figure 4B α-tubulin

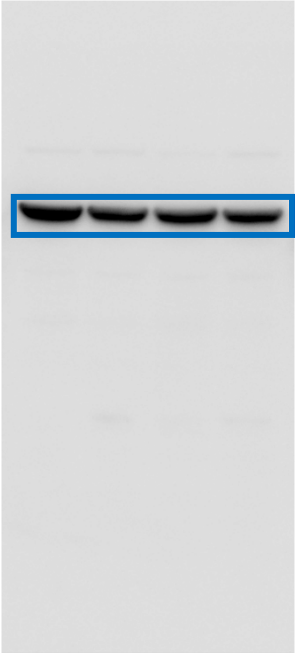

Figure 4D Mhc

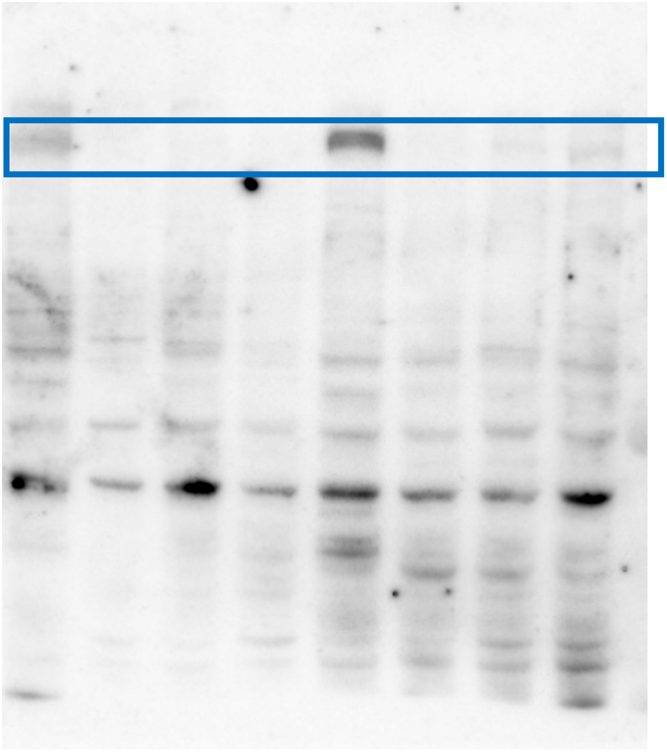

Figure 4D Pgam2

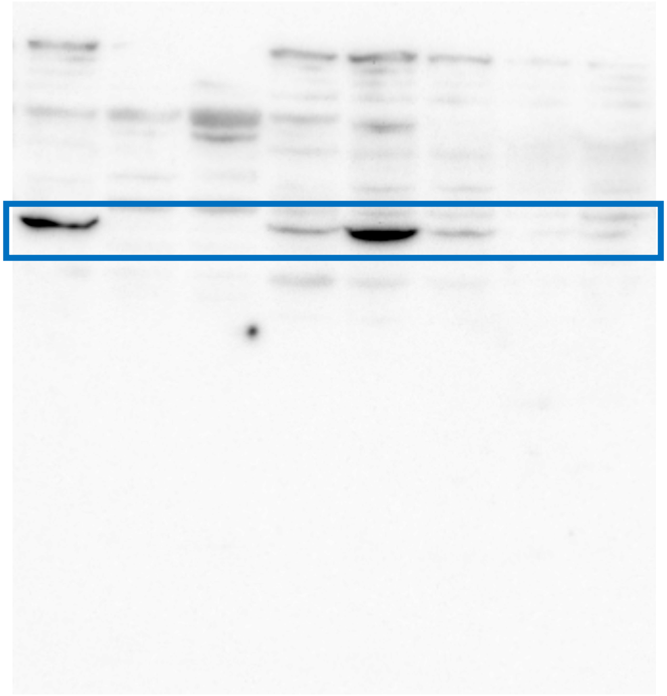

Figure 4D α-tubulin

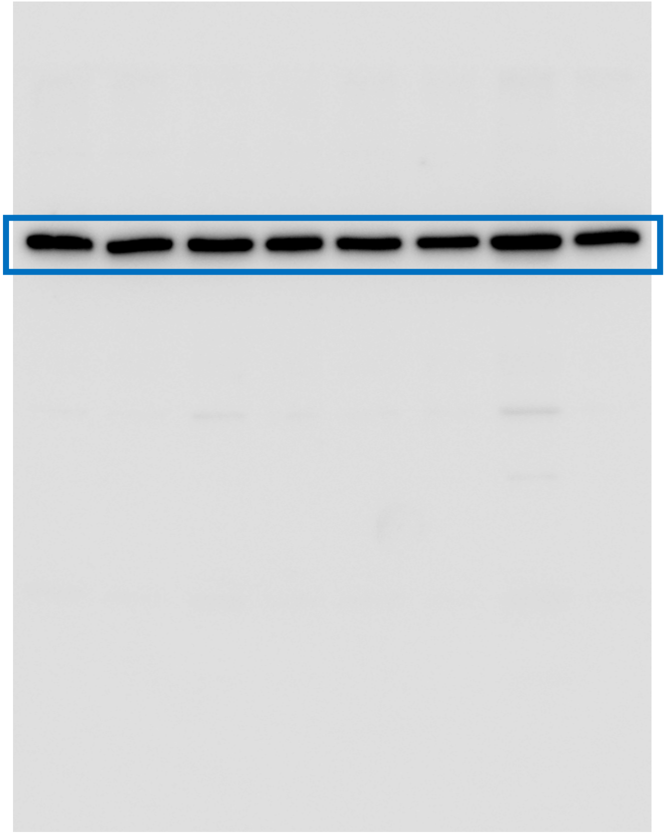

Figure 4H Pgam1

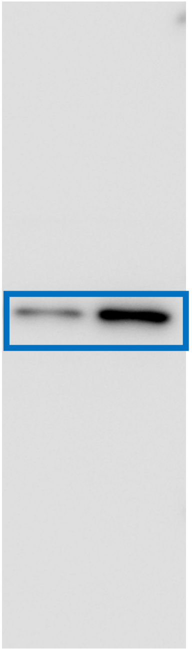

Figure 4H Phospho-Rb

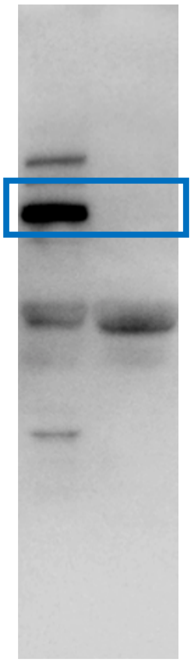

Figure 4H Rb

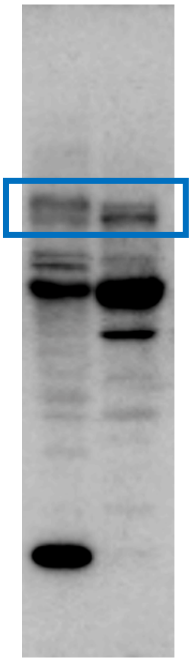

Figure 4H Pparγ

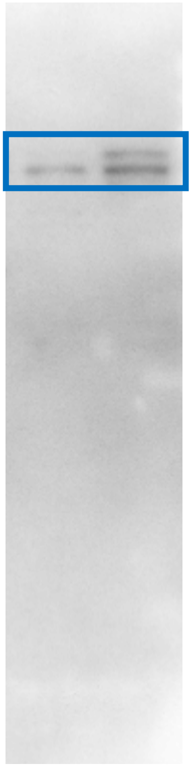

Figure 4I Pgam1

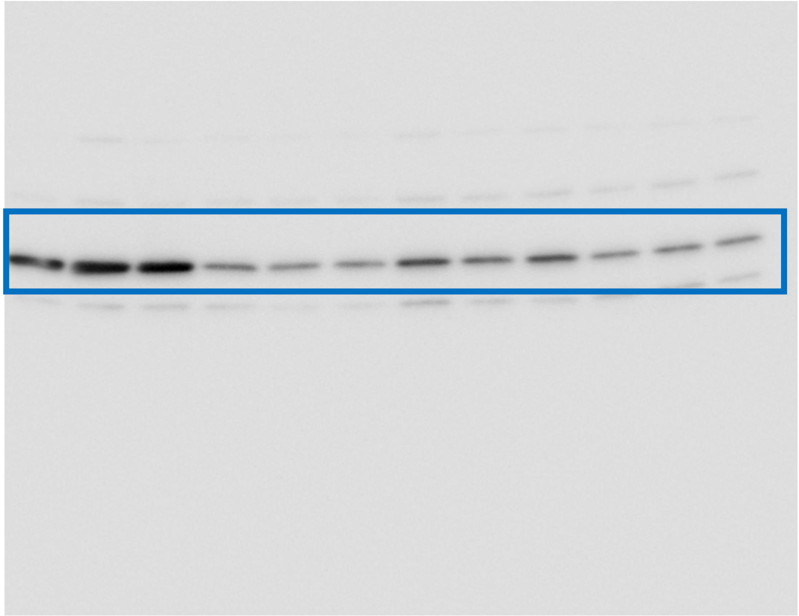

Figure 4I Pparγ

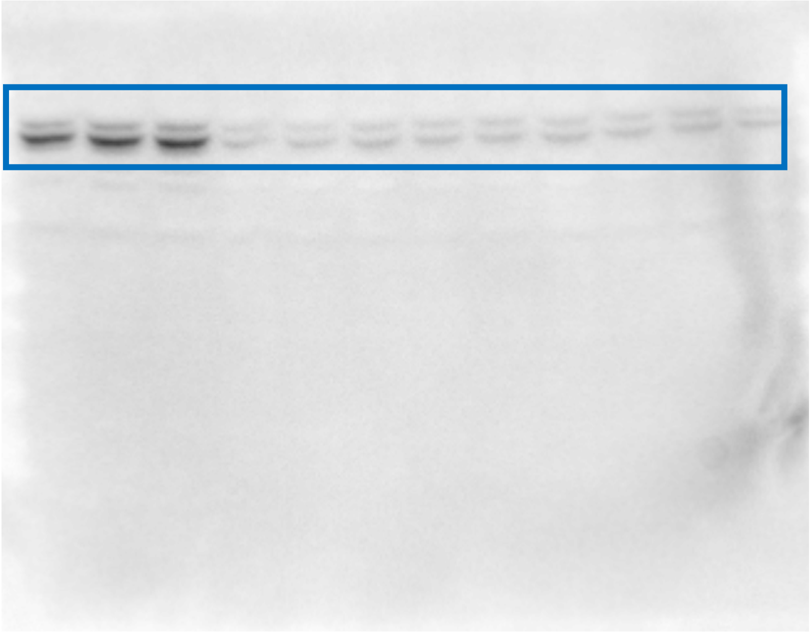

Figure 4I α-tubulin

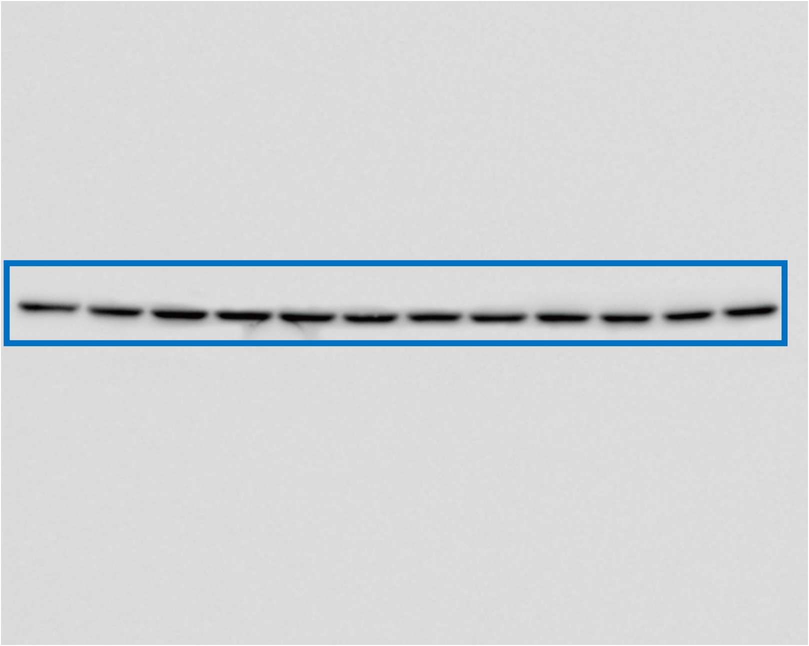

Figure 4K Pgam1

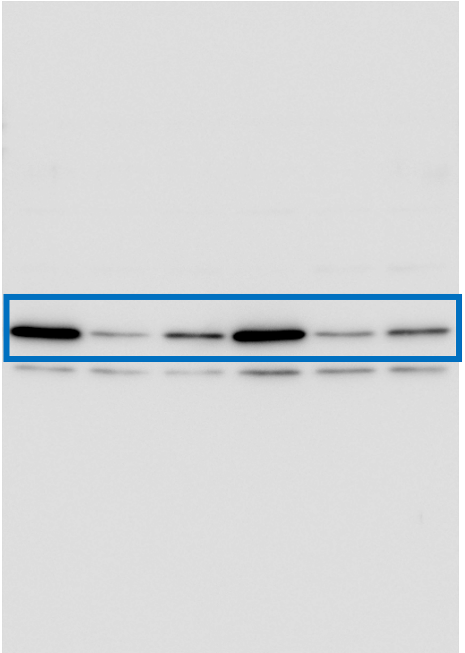

Figure 4K Pparγ

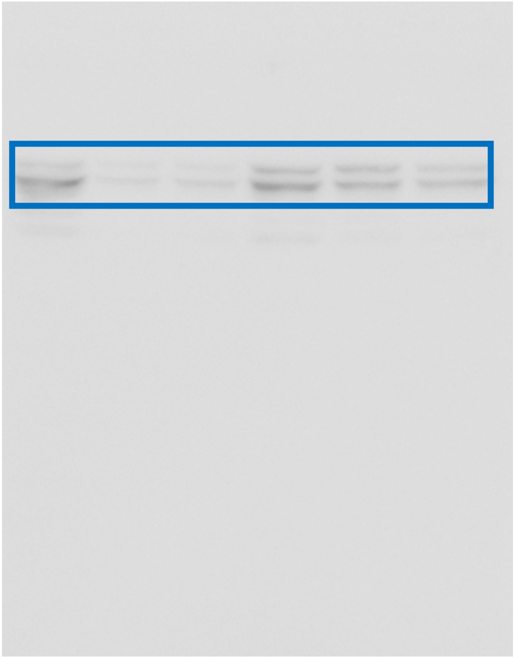

Figure 4K α-tubulin

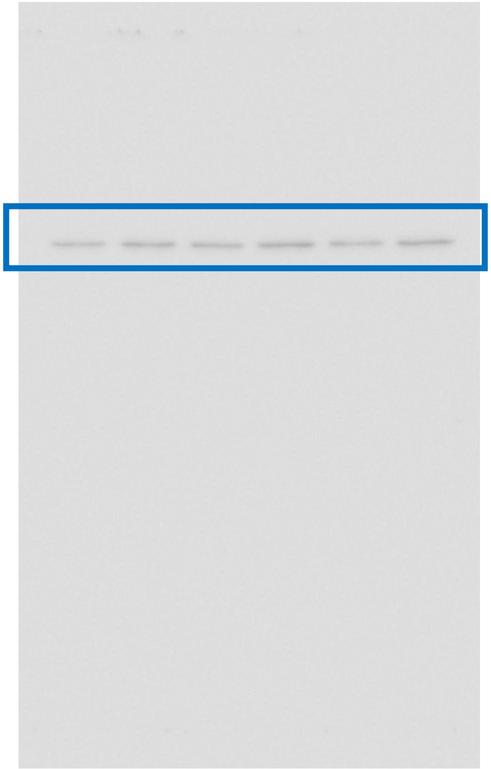

Figure 4K Pgam2

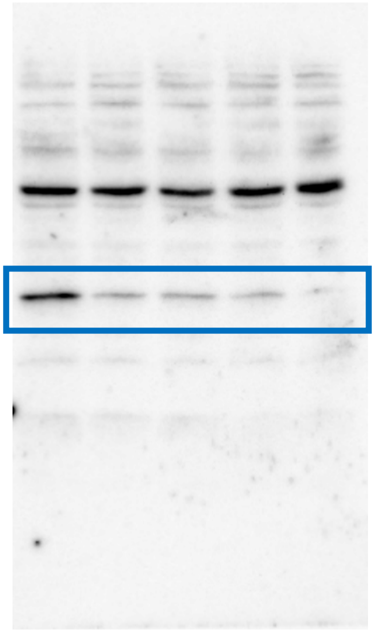

Figure 4K Mef2a

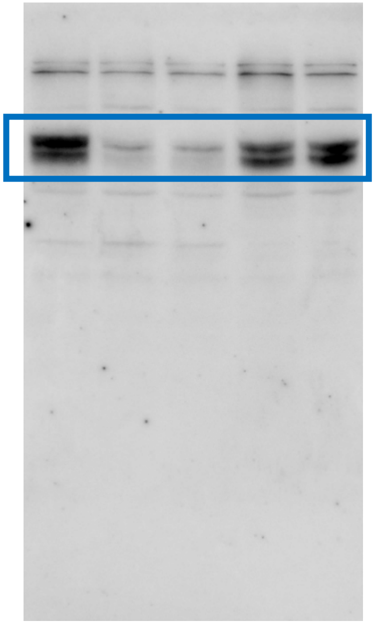

Figure 4K Mef2d

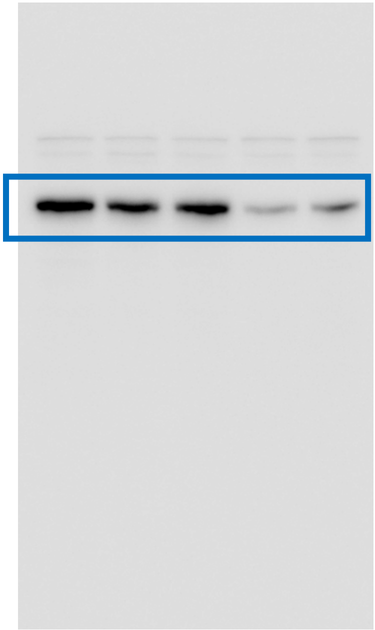

Figure 4K α-tubulin

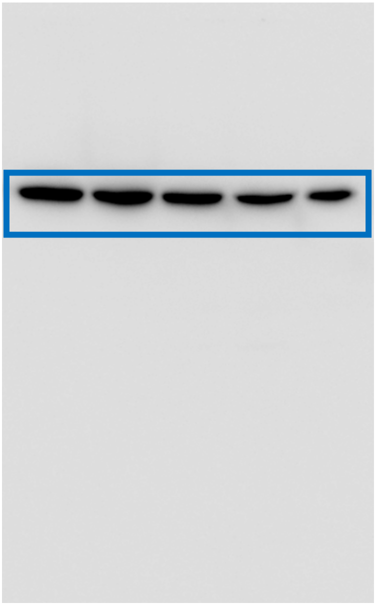

Figure 6E PGAM1

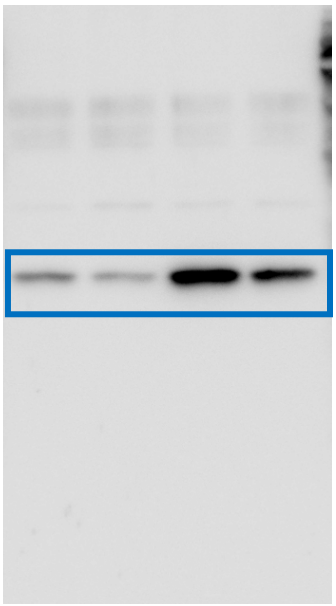

Figure 6E α-tubulin

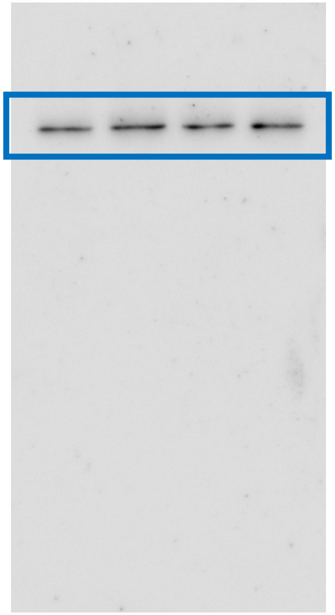

Figure 6F PGAM1

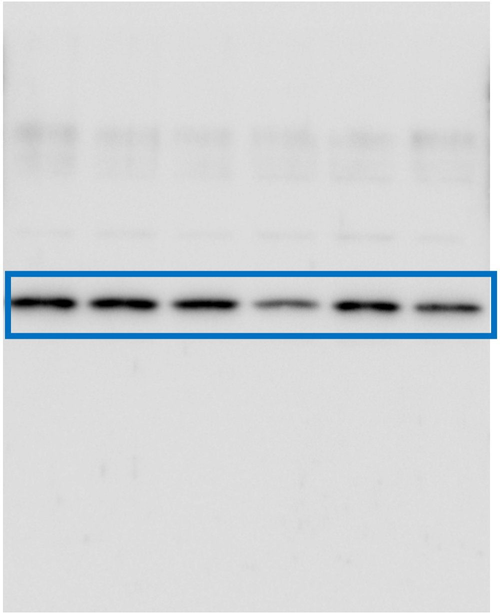

Figure 6F KDM5A

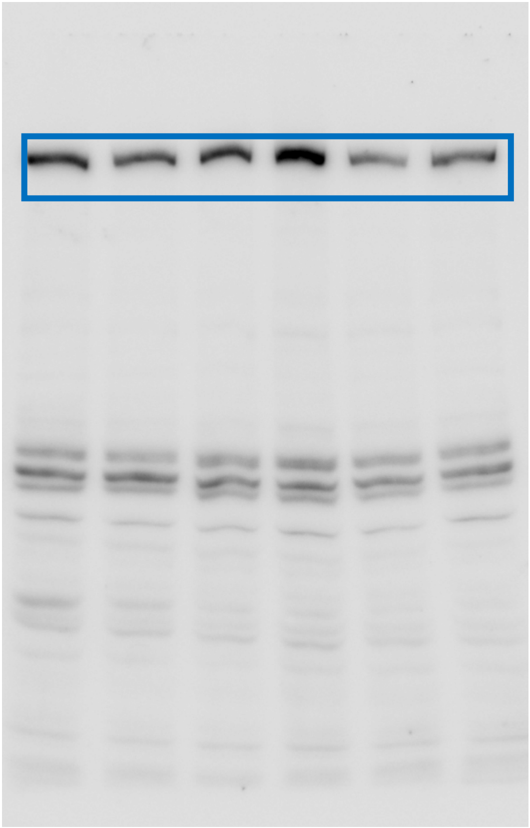

Figure 6F α-tubulin

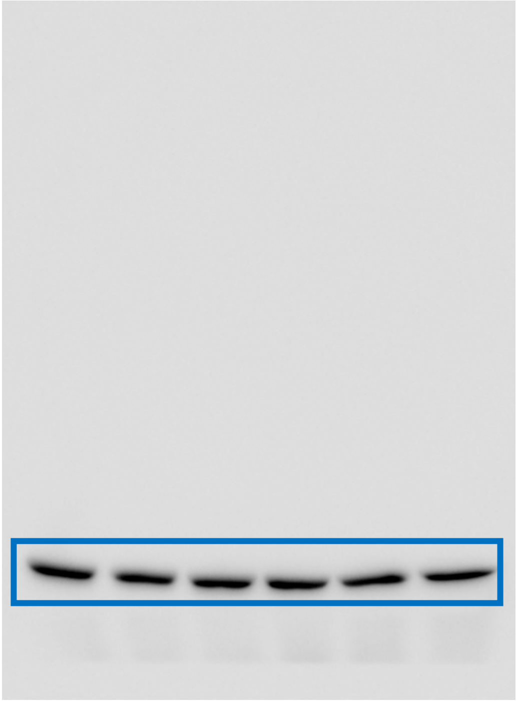

Supplement: Supplementary file 2 — Uncropped Immunoblotting [file 41419_2025_7850_MOESM2_ESM.pdf]
